# Microfluidic‐Assisted Production of Gastro‐Resistant Active‐Targeted Diatomite Nanoparticles for the Local Release of Galunisertib in Metastatic Colorectal Cancer Cells

**DOI:** 10.1002/adhm.202202672

**Published:** 2022-12-11

**Authors:** Chiara Tramontano, João Pedro Martins, Luca De Stefano, Marianna Kemell, Alexandra Correia, Monica Terracciano, Nicola Borbone, Ilaria Rea, Hélder A. Santos

**Affiliations:** ^1^ Institute of Applied Sciences and Intelligent Systems Unit of Naples National Research Council Naples 80131 Italy; ^2^ Department of Pharmacy University of Naples Federico II Naples 80131 Italy; ^3^ Drug Research Program Division of Pharmaceutical Chemistry and Technology Faculty of Pharmacy University of Helsinki Helsinki FI‐00014 Finland; ^4^ Department of Chemistry University of Helsinki Helsinki FI‐00014 Finland; ^5^ Department of Biomedical Engineering University Medical Center Groningen University of Groningen Groningen 9713 AV The Netherlands; ^6^ W.J. Kolff Institute for Biomedical Engineering and Materials Science University Medical Center Groningen University of Groningen Groningen 9713 AV The Netherlands

**Keywords:** colorectal cancer, diatomite nanoparticles, galunisertib, microfluidics, oral drug delivery

## Abstract

The oral route is highly desirable for colorectal cancer (CRC) treatment because it allows concentrating the drug in the colon and achieving a localized effect. However, orally administered drugs are often metabolized in the liver, resulting in reduced efficacy and the need for higher doses. Nanoparticle‐based drug delivery systems can be engineered to prevent the diffusion of the drug in the stomach, addressing the release at the target site, and enhancing the efficacy of the delivered drug. Here, an orally administrable galunisertib delivery system is developed with gelatin‐covered diatomite nanoparticles targeting the ligand 1‐cell adhesion molecule (L1‐CAM) on metastatic cells, and further encapsulated in an enteric matrix by microfluidics. The gastro‐resistant polymer protects the nanoparticles from the action of the digestive enzymes and allows for a sustained release of galunisertib at the intestinal pH. The efficacy of antibody–antigen interactions to drive the internalization of nanoparticles in the targeted cells is investigated in CRC cells expressing abnormal (SW620) or basal levels (Caco‐2, HT29‐MTX) of L1‐CAM. The combination of local drug release and active targeting enhances the effect of the delivered galunisertib, which inhibits the migration of the SW620 cells with greater efficiency compared to the free drug.

## Graphical abstract

The oral administration of galunisertib‐loaded nanoparticles is the key to treating colorectal cancer locally. Here, diatomite nanoparticles loaded with galunisertib are functionalized with the anti‐L1‐CAM antibody targeting metastatic colon cancer cells and encapsulated in a gastro‐resistant matrix by microfluidics. Both the local drug release and targeted internalization improve the ability of galunisertib to inhibit the migration of metastatic cells.

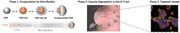

## 1 Introduction

Oral administration is not only the easiest and most tolerated drug delivery route but also the most common way to treat colon diseases locally, and thus represents an attractive approach for the treatment of colorectal cancer (CRC).^[^ ^1^ ^]^ Emerging food and drug administration (FDA)‐approved drugs inhibiting the migration and invasion of CRC cells have been brought into preclinical and clinical trials to improve the survival time of patients.^[^ ^2^, ^3^ ^]^ Among them, the small ATP‐mimetic transforming growth factor‐*β* (TGF‐*β*) inhibitor galunisertib (LY 2 157 299) has been orally administered both in monotherapy and in combination with standard antitumor regimens in Phase 2 clinical trials.^[^ ^4^ ^]^ The TGF‐*β* receptor is often overexpressed in CRC and promotes metastases by regulating cell adhesion, motility, and extracellular matrix (ECM) composition.^[^ ^5^, ^6^, ^7^ ^]^ Despite the encouraging preclinical results, the clinical translation of galunisertib calls for multiple dosing strategies due to first‐pass effects and drug metabolization, with consequent increasing side effects.^[^ ^8^ ^]^ Nanoparticles (NPs) have been providing new tools for delivering drugs with otherwise fast metabolization rates, improving drug efficacy, and enabling their release to the target site.^[^ ^9^ ^]^ In particular, inorganic porous drug delivery systems, such as silica NPs, have been shown to protect drugs from metabolizing enzymes and proteases, improve their oral bioavailability and residence time, and overall, enhance their therapeutic effect.^[^ ^10^ ^]^ However, even though cancer treatment has benefited from the NP‐based formulations that have reached the market, only a few studies have investigated the delivery of galunisertib via NPs.^[^ ^11^, ^12^ ^]^ Therefore, the potential of galunisertib‐loaded NPs for the oral treatment of CRC remains poorly explored. Our group demonstrated that the loading of galunisertib in gelatin‐modified diatomite NPs (DNPs) enhances the antimetastatic effect of the drug in CRC cells due to a matrix metalloproteinase‐triggered release from the gelatin matrix.^[^ ^13^ ^]^ DNPs are inorganic NPs obtained from the porous siliceous skeletons of diatoms, being thus made of biocompatible porous silica, which is useful for efficient drug entrapment and delivery to cells.^[^ ^14^, ^15^, ^16^, ^17^, ^18^, ^19^, ^20^ ^]^ The oral administration of gelatin‐covered DNPs (DNPs‐Gel) is, however, a hard task due to the degradation of gelatin by digestive enzymes in the gastrointestinal (GI) tract, which would cause the undesired release of galunisertib. The development of a gastro‐resistant coating on DNPs‐Gel by microfluidics could overcome gelatin degradation in the stomach and help address the drug release in the colon. Microfluidics has gained increasing relevance for the production of advanced drug delivery systems, promoting the translation of NPs from research to clinical applications.^[^ ^21^, ^22^ ^]^ Compared to bulk mixing processes, which often result in polydisperse NPs, microfluidics enables the precise encapsulation of NPs due to well‐controlled flow rates of the gastro‐resistant polymer solution and NPs inside micrometer‐sized channels.^[^ ^23^, ^24^, ^25^ ^]^ This cutting‐edge technology makes it possible to encapsulate nanocarriers with high batch‐to‐batch reproducibility and gastro‐resistant properties, protecting gelatin from degradation and enabling the release of galunisertib in the target site. The DNPs‐Gel also hold great promise for CRC treatment due to the possibility of modifying the gelatin coverage with an antibody targeting the ligand‐1 cell adhesion molecule (L1‐CAM) on cancer cells. L1‐CAM is a transmembrane protein involved in the dynamic process of cancer metastasis and invasion of a secondary tumor site, and therefore, is often overexpressed in CRC.^[^ ^26^ ^]^ Recent findings suggest that this antigen helps CRC cells adhere and spread on the surface of blood capillaries, initiating the metastatic outgrowth in perivascular sites.^[^ ^27^, ^28^ ^]^ The specific interactions of antibody‐modified DNPs‐Gel with CRC cells expressing high levels of L1‐CAM would prevent off‐target effects caused by unspecific DNP uptake and galunisertib release.

Here, inspired by the need for a CRC‐targeted therapy, we fabricate a galunisertib delivery system for oral administration made of DNPs‐Gel modified with an anti‐L1‐CAM antibody (DNPs‐Gel‐Ab) and encapsulated in hydroxypropyl methylcellulose acetate succinate (HPMC‐AS, herein abbreviated as HPMC) by microfluidics. We use an in‐house glass‐capillary microfluidic nanoprecipitation technique to achieve a homogeneous and controlled encapsulation of the DNPs‐Gel‐Ab in the gastro‐resistant HPMC matrix (Encapsulated‐DNPs).^[^ ^29^, ^30^ ^]^ The proposed microfluidic encapsulation enables considering, for the first time, the oral administration of DNPs, which is expected to overcome challenges associated with their injection, such as extravasation from blood to tumor, blood clearance, and systemic toxicity.^[^ ^31^ ^]^ To further decrease side effects, we propose active‐targeted encapsulated‐DNPs capable of selectively binding to cells overexpressing the L1‐CAM and increasing the local concentration of released galunisertib. By concentrating the delivered drug in metastatic CRC cells, our nanocarrier is expected to reduce the dose of the drug required to inhibit cell migration, thereby decreasing the likelihood of adverse side effects. To analyze the efficiency of the active‐targeted approach in our formulation, we investigate and quantify the uptake of the DNPs in three CRC cell lines expressing high (SW620) or basal (Caco‐2, HT29‐MTX) levels of the surface antigen L1‐CAM. Finally, we study the ability of galunisertib released by the encapsulated‐DNPs to inhibit the TGF‐*β*‐mediated migration of SW620 metastatic cancer cells.

## 2 Results and Discussion

### 2.1 Production and Physicochemical Characterization of NPs

**Scheme 1:**
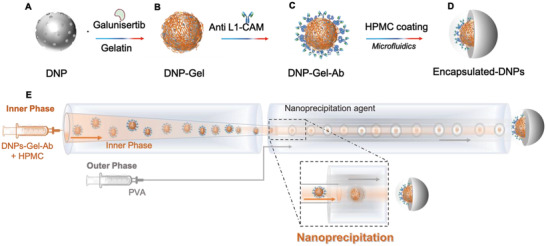
Schematic representation of the developed NPs and their encapsulation via microfluidics. A,B) Galunisertib loading and coverage of DNPs with gelatin. C) Attachment of the anti‐L1‐CAM antibody to the DNP‐Gel. D) Encapsulation of DNPs‐Gel into the enteric polymer. E) Design of the microfluidic channel, which is composed of an outer capillary, in which the nanoprecipitation agent polyvinyl alcohol (PVA) is pumped, and an inner capillary through which the dispersion of DNPs ‐Gel‐Ab and HPMC is injected.

The DNPs serving as drug nanocarriers were functionalized and encapsulated as sketched in **Scheme** **1** to fabricate a gastro‐resistant oral formulation capable of delivering galunisertib specifically to CRC cells. First, the diatomite earth powder, composed of algae skeletons known as frustules, was dispersed in ethanol and ultrasonicated for 160 h to reduce the size of the frustules at the nanoscale level. DNPs were centrifuged, collected and purified with a mixture of sulfuric acid (H_2_SO_4_) and hydrogen peroxide (H_2_O_2_) to purge the organic contamination, and with hydrochloric acid (HCl) to remove metal residues. Then, they were washed with Milli‐Q H_2_O to remove traces of the acid treatments and aminosilanized with 10% v/v 3‐aminopropyltriethoxysilane (APTES) solution (referred to as “DNPs” here) as previously described.^[^ ^12^, ^13^ ^]^ The produced DNPs were loaded with galunisertib and further covered by a layer of crosslinked gelatin (Scheme 1A,B). Then, the anti‐L1‐CAM antibody was bound to the gelatin layer via protein A, and the DNPs‐Gel‐Ab were encapsulated in HPMC by microfluidics for oral administration (Scheme 1C,D).

Particle size is an important parameter for the choice of the appropriate administration route. The intravenous injection is generally suggested for NPs smaller than 150 nm but it is not suitable for DNPs with an average size of 400 nm.^[^ ^32^ ^]^ The most suitable administration for this system, instead, is represented by the oral route. It has been demonstrated that the small intestine epithelium can easily uptake particles 1 µm in size, offering the highest level of tissue uptake for NPs with an average size of 200–500 nm.^[^ ^33^ ^]^ Particles with smaller sizes are easier to be metabolized outside the body, reducing the retention time of the delivered drug in the tumor site.^[^ ^34^, ^35^ ^]^ Bigger NPs, instead, are more likely to be retained upon oral administration, thus increasing the drug availability at the target site.

**Figure 1:**
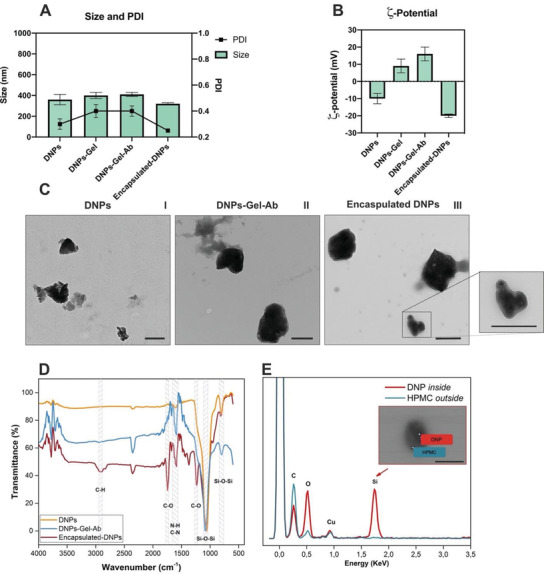
Characterization of the physicochemical properties of the developed DNPs: A) Size and PDI, and B)  *ζ*‐potential. Results are expressed as mean ± s.d. (*n* ≥ 3). C) TEM images of DNPs (I), DNPs‐Gel‐Ab (II), and encapsulated‐DNPs (III). The scale bars are 500 nm. D) ATR‐FTIR spectra of the DNPs, DNPs‐Gel‐Ab, and encapsulated‐DNPs. E) Elemental composition of encapsulated‐DNPs by EDX analysis. The scale bar in the inset is 500 nm.

The DNPs produced by ultrasonication and purification approaches had an average size of 360 ± 50 nm and a surface charge of −10 ± 3 mV due to the negatively charged hydroxyl groups (—OH^−^) (**Figure** **1**A). The gelatin layer induced a slight increase in the DNPs’ size (about 40 nm) and provided the surface with positive amino groups (—NH_2_) useful for further functionalization steps. Gelatin was crosslinked via the formation of an intramolecular peptide bond using 1‐ethyl‐3‐[3‐dimethylaminopropyl]carbodiimide‐hydrochloride and *N*‐hydroxysuccinimide (EDC/NHS) chemistry. The coating with crosslinked gelatin increased the PDI of the DNPs from 0.30 ± 0.04 to 0.40 ± 0.05 as expected due to the low degree of control over the bulk mixing process of gelatin with the DNPs. The encapsulation of DNPs in gelatin and further crosslinking could also be carried out in a microfluidic platform with three inlets, in which the dispersion of DNPs and gelatin, antisolvent solution, and crosslinking agents are pumped separately.^[^ ^36^ ^]^ However, the chemical crosslinking by EDC/NHS is generally a time‐consuming step in controlled pH conditions. Long‐term reactions may increase the likelihood of particle deposition on the bottom of the microfluidic channel and contribute to its complete or partial clogging. Alternative crosslinking techniques, such as cooling, photopolymerization, or ionic bonding can be performed in microfluidic channels.^[^ ^37^, ^38^ ^]^ The chemical crosslinking of gelatin, instead, was shown to be successfully achieved by bulk mixing, allowing for the development of a galunisertib delivery system with pH and enzyme responsive features.^[^ ^19^ ^]^ As a result of these aspects, herein, the gelatin coating on the DNPs and further crosslinking was achieved by bulk mixing, whereas the encapsulation in HPMC was performed by microfluidics.

For binding the anti‐L1‐CAM antibody to the NPs, we attached protein‐A (Pr‐A) to the —NH_2_ groups of gelatin by EDC/NHS and then incubated Pr‐A‐modified DNPs‐Gel with the antibody. Protein A helped the antibody orient itself and bind to the DNPs’ surface through the fragment crystallizable region (Fc), thus making the fragment antigen‐binding (Fab) accessible to the antigen.^[^ ^39^ ^]^ The DNPs‐Gel‐Ab showed an average size of 410 ± 20 nm and a surface charge of 16 ± 4 mV (Figure 1B), which later on favored the interaction with the negatively charged functional groups of the HPMC. This enteric polymer is a regular excipient of solid dosage forms, widely used as a coating material for orally‐administered formulations to protect the drug from the harsh acidic conditions of the stomach.^[^ ^40^ ^]^ For the encapsulation of DNPs‐Gel‐Ab in HPMC, we used a microfluidic platform consisting of inner and outer capillaries aligned in a co‐flow geometry (Scheme 1E). The inner phase was composed of a dispersion of DNPs‐Gel‐Ab and HPMC in 50:50 ethanol: water and was pumped into the inner capillary, whereas the nanoprecipitation agent polyvinyl alcohol (PVA) was injected from the outer capillary as previously described.^[^ ^30^ ^]^ The inner and outer phases flowed in the same direction to create a 3D coaxial flow, in which the aqueous PVA solution helped the precipitation of HPMC and the entrapment of DNPs‐Gel‐Ab in the polymer matrix. Compared to the conventional bulk mixing methods, this technology allowed a precise encapsulation of DNPs‐Gel‐Ab due to the possibility of controlling volumes and flow rates of the two phases. The encapsulation was confirmed by a decrease in the size and PDI of the formulation, which turned from 410 ± 20 to 320 ± 10 nm and from 0.40 ± 0.05 to 0.25 ± 0.01 nm, respectively, due to the improved morphology and stability provided by the homogeneous polymeric coating. The controlled encapsulation provided the system with a less irregular shape (Figure 1C‐III), which, in turn, improved the PDI. The nanoprecipitation of HPMC was also confirmed by the negatively charged surface of encapsulated‐DNPs, which turned from 14 ± 4 to −20 ±1 mV due to the —COOH of the polymer matrix. The consistent negative surface charge of the encapsulated DNPs increased the internal repulsion forces between encapsulated‐DNPs and avoided their aggregation. The HPMC was first absorbed on the DNPs‐Gel‐Ab flowing through the inner capillary, and, later on, the antisolvent PVA solution caused the nanoprecipitation of HPMC and the formation of a controlled coating around encapsulated‐DNPs. The likelihood of NPs’ aggregation with this technique is lower than with the bulk mixing method, due to the continuous adjustment of the reaction conditions over the mixing rates of reagents.^[^ ^25^ ^]^ The increased stability of encapsulated‐DNPs was demonstrated in aqueous 2‐(*N*‐morpholino)ethanesulfonic acid hemisodium (MES) pH 4.5 up to 36 h (Figure S1, Supporting Information).

Transmission electron microscopy (TEM) imaging showed that the ultrasonication approach produced DNPs with irregular size and shape (Figure 1C‐I), which are the expected features of this type of NPs. However, the uneven surface of DNPs is not likely to affect their interactions with the cells, as previously reported.^[^ ^14^ ^]^ The gelatin was absorbed into DNPs creating a thin layer that made the surface of DNPs smoother (Figure 1C‐II). Since the bulk mixing of gelatin and DNPs offered a low control over the process, free gelatin not embedding the DNPs‐Gel‐Ab can be seen on the TEM grid. However, after encapsulation in HPMC, the formulation showed a higher homogeneity in morphology, as a result of the well‐controlled microfluidic process (Figure 1C‐III). The improved morphology provided by the microfluidic entrapment was in line with the decreased PDI value obtained with the DLS. The controlled nanoprecipitation of the polymer around NPs led to the formation of a distinguishable in‐out structure made of silica and polymer (Figure S2, Supporting Information). The DNPs were further characterized by attenuated total reflectance Fourier transform infrared (ATR‐FTIR) and energy dispersive X‐ray (EDX) spectroscopy at different steps of functionalization (Figure 1D,E). The FTIR spectrum of DNPs showed two characteristic absorption bands at 1075 and 800 cm^−1^ related to the Si‐O‐Si groups, and the band at 1600 cm^−1^ ascribed to the aminosilanization (Figure 1D).^[^ ^13^ ^]^ This band increased in DNPs‐Gel‐Ab and can be related to both the crosslinked gelatin (C—N) and antibody (N—H) labeling, which resulted in a positive surface charge of DNPs‐Gel‐Ab (Figure 1B).^[^ ^41^ ^]^ The C—O stretch at 1740 and 1230 cm^−1^, and the C—H band at 2927 cm^−1^ in the spectrum of encapsulated‐DNPs confirmed the presence in the sample of an organic matrix of HPMC in the sample.^[^ ^30^ ^]^ The proof that DNPs‐Gel‐Ab were encapsulated in HPMC was obtained by EDX elemental analysis (Figure 1E). We analyzed both the inner DNPs‐Gel‐Ab and the polymer matrix surrounding them. The silicon (Si) peak appeared in the spectrum when we analyzed the content of encapsulated‐DNPs, that is the DNP‐Gel‐Ab, whereas only the carbon (C) peak was detected when we investigated the polymer matrix of HPMC. Overall, the EDX analysis confirmed that the DNPs‐Gel‐Ab were efficiently entrapped in the HPMC matrix by creating an in‐out structure, in which the inner part was composed of the DNPs‐Gel‐Ab and the outer of the HPMC polymer.

### 2.2 Drug Loading, Release, and Dissolution Studies of the Encapsulated‐DNPs

The transit through the GI tract is influenced by different parameters, including the residence time, pH, and presence of digestive enzymes.^[^ ^42^ ^]^ The gastric time can vary from patient to patient and ranges from 0 to 2 h, whereas the transit in the small intestine is considered relatively constant (4 h). The colonic transit time can be, instead, highly variable and affected by the local disease, with ranges from 1 to 50 h.^[^ ^43^ ^]^ Therefore, to study the behavior of the encapsulated‐DNPs in the GI tract, we dispersed encapsulated‐DNPs in simulated gastric fluid (SGF) pH 1.6 with pepsin (1 mg mL^−1^) for 2 h. Then, the encapsulated‐DNPs were dispersed in fasted‐state simulated intestinal fluid (FaSSIF) pH 5.5 enriched with trypsin (0.06 mg mL^−1^). The concentration of trypsin along the small and large intestine can vary significantly from 0.03 to 0.13 mg mL^−1^ from the upper part of the bowel to the lower duodenum.^[^ ^44^ ^]^ Due to these fluctuations, we used a mean value of trypsin concentration (0.06 mg mL^−1^) for mimicking the passage of the encapsulated‐DNPs through the intestine, as also reported elsewhere.^[^ ^12^ ^]^ To simulate the transit through the colon, the encapsulated‐DNPs were then dispersed in FaSSIF pH 8.0 supplemented with trypsin for 2 h.^[^ ^45^ ^]^ Before the galunisertib release studies, we investigated the drug loading capacity of DNPs‐Gel‐Ab and after the encapsulation process (Figure S3, Supporting Information). To this aim, the DNPs‐Gel‐Ab were dispersed in 1 mL of PBS solution supplemented with trypsin to degrade the gelatin matrix, then they were centrifuged and supernatants were analyzed by reversed‐phase high‐performance liquid chromatography (RP‐HPLC). The encapsulated‐DNPs, instead, were first dispersed in 70% ethanol to dissolve the polymer matrix, and then suspended in a PBS solution enriched with trypsin to favor the enzymatic degradation of gelatin.

The DNPs‐Gel‐Ab and encapsulated‐DNPs showed a very similar galunisertib loading capacity of 4.5% ± 0.3% and 4.0% ± 0.2%, respectively (Figure S3, Supporting Information). The decreased loading capacity after encapsulation can be explained by the loss of the drug that was loosely absorbed on the surface of DNPs‐Gel‐Ab and, thus, released during the encapsulation process. The loading capacity of encapsulated‐DNPs is consistent with the loading efficiencies reported for most existing nanocarriers with a similar porosity via the immersion method.^[^ ^46^ ^]^ Higher loading efficiencies were achieved by increasing the surface area of NPs, which is a non‐tuneable parameter for naturally porous DNPs.^[^ ^47^, ^48^ ^]^ We reported in a previous work that DNPs‐Gel having a lower galunisertib loading capacity of 2% ± 0.4% inhibited the metastatic signaling in CRC efficiently.^[^ ^13^ ^]^ Here, a higher loading capacity of 4% ± 0.2% was achieved by increasing the concentration of gelatin in the outer shell, as suggested by previous results.^[^ ^12^ ^]^ Due to the improved loading capacity of encapsulated‐DNPs, a non‐toxic concentration of NPs can be administered to inhibit migration with greater efficiency than the free galunisertib.

**Figure 2:**
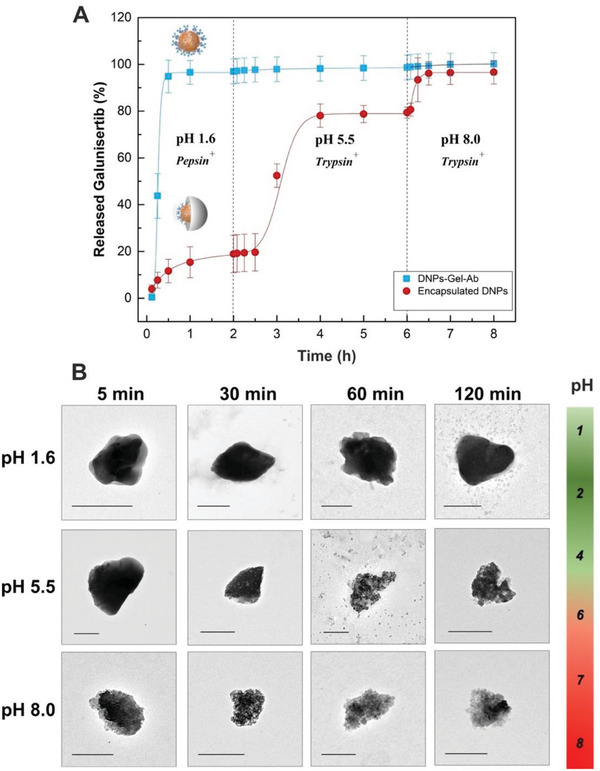
Drug release and dissolution profile studies. A) The drug release studies of the DNPs‐Gel‐Ab (blue line) and encapsulated‐DNPs (red line) in SGF at pH 1.6 and FaSSIF at pH 5.5 and 8.0 were investigated by RP‐HPLC. Results are expressed as mean ± s.d. (*n* ≥ 3). B) TEM images of the encapsulated‐DNPs after incubation in different pH environments. The scale bars are 300 nm.

Due to the diverse external shells, the drug release profiles of the DNPs‐Gel‐Ab and encapsulated‐DNPs were very different and followed distinct release kinetics (**Figure** **2**A). For the DNPs‐Gel‐Ab, we observed a burst release of galunisertib at pH 1.6 within 30 min, due to both the acidic microenvironment and the presence of pepsin. Gelatin is a pH‐responsive polymer unfolding at pH < 5 and can be degraded by pepsin on the N‐terminal residues. Therefore, the gelatin matrix in the DNPs‐Gel‐Ab got unfolded in the SGF buffer, and, as a consequence, the chains got accessible to the pepsin, which degraded the polymer and favored the release of 100% of galunisertib in <30 min.^[^ ^49^ ^]^ For the encapsulated‐DNPs, instead, only 20% of galunisertib was released after 2 h at pH 1.6, whereas a consistent amount of the drug (≈80%) was released in FaSSIF pH 5.5. Here, the gastro‐resistant HPMC matrix protected the gelatin layer covering the surface of DNPs from the acidic pH, making the N‐terminal residues of gelatin inaccessible to pepsin degradation. As soon as the polymer started degrading in FaSSIF pH 5.5, the gelatin on the DNPs‐Gel‐Ab was degraded by trypsin and galunisertib was gradually released within 4 h. Then, when the encapsulated‐DNPs were dispersed in FaSSIF pH 8.0 supplemented with trypsin, the HPMC was completely dissolved, gelatin was digested by trypsin, and 100% of the drug was released at the colon pH. The developed oral formulation was designed to release galunisertib upon dissolution of the enteric coating at the intestinal pH. During the passage between the intestine and colon, however, the HPMC dissolution exposes the inner DNPs‐Gel‐Ab to the environment and the drug release id triggered by gelatin enzymatic digestion. Without the gelatin shell, however, galunisertib would be released from the DNPs in the intestine after dissolution of the HPMC. The gelatin layer, therefore, played a crucial role in the formulation as it controlled the release of galunisertib at intestinal pH and ensured its accumulation in the colon while avoiding burst release. The drug release studies herewith suggest that the critical factors controlling the release of galunisertib from encapsulated‐DNPs were the dissolution of both the enteric polymer and gelatin.

To investigate the polymer dissolution, we analyzed the morphology of the encapsulated‐DNPs after being dispersed in SGF at pH 1.6 and FaSSIF at pH 5.5 and 8.0 by TEM (Figure 2B). The TEM images confirmed that the enteric polymer remained intact when encapsulated‐DNPs were dispersed at pH 1.6 for 2 h. As a result of the gastro‐resistant features of the HPMC in these pH conditions, only 20% of the encapsulated drug was released within 2 h (Figure 2A). The dissolution of HPMC occurred rapidly at pH 5.5 since the polymer was completely dissolved after 10 min in the tested pH conditions, exposing the inner DNPs. However, even if the dissolution of the enteric polymer was very fast at pH 5.5, galunisertib was gradually released within 4 h thanks to the crosslinked gelatin matrix. The TEM images suggested that the HPMC matrix dissolved immediately at pH 8.0 since the enteric matrix was no longer detectable after 5 min and only DNPs could be seen on the TEM grid. Overall, the microfluidic encapsulation in HPMC makes the DNPs‐Gel‐Ab resistant to the harsh conditions of the stomach after oral administration, allowing for the release of galunisertib in the small intestine and colon.

### 2.3 Quantification of L1‐CAM Expression on Caco‐2, HT29‐MTX, and SW620 Cells and Cell Viability Studies

**Figure 3:**
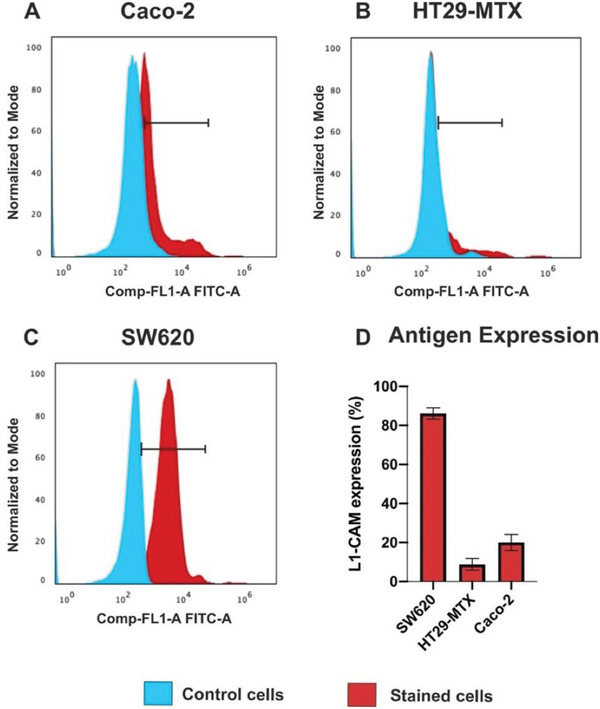
Quantification of L1‐CAM expression in A) Caco‐2, B) HT29‐MTX, and C) SW620 cells by FACS. Cells were incubated with the primary and secondary antibodies and sorted by fluorescence in the APC channel. D) The statistical analysis is reported as mean ± s.d. (*n* ≥ 3).

The L1‐CAM is a glycoprotein involved in cancer development and associated with metastases and poor prognosis.^[^ ^28^ ^]^ The expression of L1‐CAM is frequently increased in metastasis‐initiating CRC cells and promotes their migration and invasion of the liver.^[^ ^50^ ^]^ Therefore, before encapsulation, our nanosystem was modified with the anti‐L1‐CAM antibody to address the interactions of the NPs with the metastatic CRC cells overexpressing the antigen L1‐CAM. Considering that the developed formulation was envisaged for the treatment of colon cancer, we selected three CRC cell lines expressing different levels of the L1‐CAM (SW620, Caco‐2 and HT29‐MTX) for the in vitro studies. The SW620 cell line constitutes a unique model for studying the later stages of CRC since it was derived from a patient affected by Duke's stage B colon carcinoma with liver and lymph node metastases.^[^ ^51^ ^]^ The Caco‐2 and HT29‐MTX cells were isolated from the colon tissue of a patient with colorectal adenocarcinoma, and they both have an epithelial morphology.^[^ ^52^, ^53^ ^]^ Moreover, Caco‐2 cells represent ≈90% of the intestinal epithelium, and the goblet‐like and mucus‐producing HT29‐MTX cells represent ≈10% of the intestinal cells.^[^ ^54^ ^]^ Therefore, both Caco‐2 and HT29‐MTX represent the most used in vitro gastrointestinal models for investigating the efficacy of oral dosage forms. Since the overexpression of L1‐CAM promotes the ETM process by which cells acquire metastatic capacities, we expected that the L1‐CAM was overexpressed in the metastatic SW620 cells, whereas Caco‐2 and HT29‐MTX exhibited basal levels of the antigen. To confirm this, we investigated the expression of the antigen in the SW620, Caco‐2, and HT29‐MTX by fluorescence‐assisted cell sorting (FACS) (**Figure** **3**). The SW620, Caco‐2, and HT29‐MTX cells were incubated with the anti‐L1‐CAM primary antibody. Then, they were washed to remove the unbound molecules, and incubated with the secondary antibody labeled with a fluorophore. Finally, the cells were washed and sorted by FACS in the allophycocyanin (APC) channel. The expression of the L1‐CAM was observed in 20% ± 4% of Caco‐2 cells and 9% ± 2% of HT29‐MTX, suggesting that the epithelial cells express a basal level of the endogenous antigen (Figure 3A,B). By contrast, as expected based on the metastatic phenotype, the L1‐CAM was expressed in 86% ± 3% of the SW620 cells. (Figure 3C). Hence, the SW620 cell line represented the ideal metastatic target to investigate the potential of our formulation, whereas Caco‐2 and HT29‐MTX cells served as the epithelial model of CRC cells.

**Figure 4:**
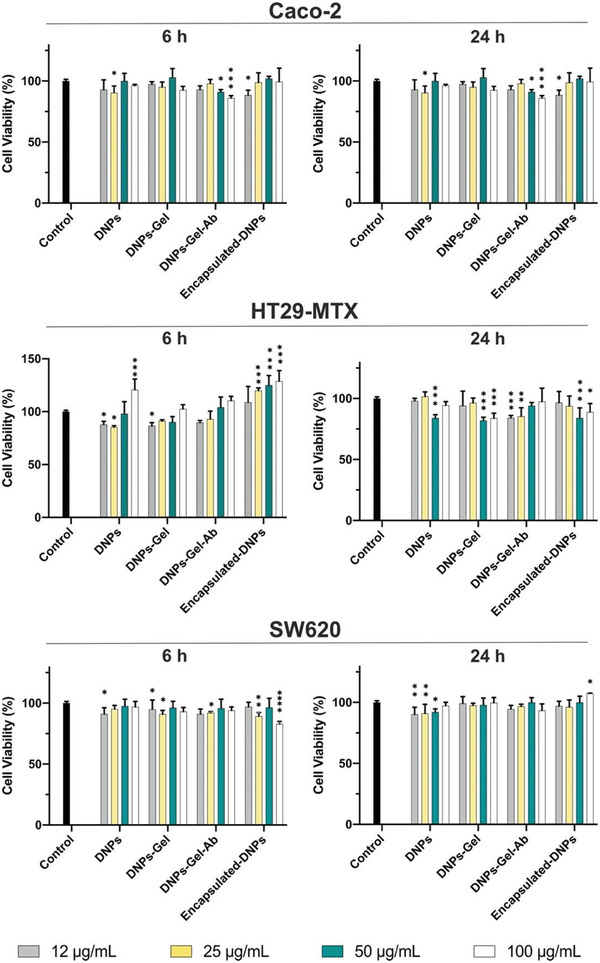
Biocompatibility studies. Cell viability (%) of Caco‐2, HT29‐MTX, and SW620 incubated with NPs at different steps of preparation (DNPs, DNPs‐Gel, DNPs‐Gel‐Ab, encapsulated‐DNPs) and concentrations ranging from 25 to 100 µg mL^−1^. Cells were incubated with HBSS−HEPES buffer (pH 7.2) as the negative control. Each data set was compared to the negative control. The level of significance was set at probabilities of * *p* < 0.05, ** *p* < 0.01, and *** *p* < 0.001. Results are expressed as mean ± s.d. (*n* ≥ 3).

After quantifying the expression of the antigen in the selected cell lines, we investigated if any of the components used in the formulation could be toxic to cells for up to 24 h. For this purpose, the SW620, Caco‐2, and HT29‐MTX cells were incubated with the DNPs at different steps of preparation (DNPs, DNPs‐Gel, DNPs‐Gel‐Ab, and encapsulated‐DNPs) at different concentrations (12, 25, 50, and 100 µg mL^−1^) and time points. The cell viability was measured by using the adenosine triphosphate (ATP)‐based luminescence assay after 6 and 24 h of incubation, corresponding to the minimum (6 h) and maximum (24 h) residence time in the GI tract, respectively, when NPs are taken up in the mucosa.^[^ ^55^ ^]^ Results showed that all the tested formulations were biocompatible at different concentrations and did not induce toxicity in the SW620, Caco‐2, and HT29‐MTX cells after 24 h of incubation (**Figure** **4**).

The cell viability of Caco‐2 and SW620 cells incubated with each formulation was over 85% after 6 and 24 h of incubation, regardless of the concentration. For HT29‐MTX, instead, fluctuations of the cell viability values were observed when the cells were incubated with the DNPs for 6 h. However, even if NPs conditioned the viability of HT29‐MTX cells within this time interval, no cytotoxicity was observed. The incubation of the goblet‐like cells with the developed DNPs at every step of preparation for a longer time (24 h) did not alter cell viability, which was higher than 80% in each tested condition. The viability of SW620, HT29‐MTX, and Caco‐2 cells was >85% after 24 h, demonstrating that none of the developed formulations inhibited cell proliferation. The encapsulated‐DNPs, corresponding to the final system, reported high cytocompatibility with both the metastatic and epithelial cell lines up to 100 µg mL^−1^ and 24 h of incubation. The transit of the developed formulation through the colon, however, may be longer than 24 and take up to 70 h, according to the weight, sex, and health conditions of patients.^[^ ^55^ ^]^ An intriguing advantage of using DNPs over organic NPs for oral administration is their thermal and chemical stability, which enables DNPs to be retained in the colon for up to 70 h. To demonstrate the safety of the developed formulation, we also investigated the cell viability of the Caco‐2, HT29‐MTX, and SW620 upon incubation with the NPs for 72 h (Figure S4, Supporting Information). The viability of the SW620 and HT29‐MTX cells was over 75% after incubation with the highest concentration (100 µg mL^−1^) of each developed formulation for 72 h. The incubation of the Caco‐2 cells with 25 µg mL^−1^ of the encapsulated‐DNPs decreased the cell viability from 100% (control cells) to 60% ± 8% (Figure S4, Supporting Information). However, the treatment of Caco‐2 with the highest concentrations of the encapsulated‐DNPs (100 µg mL^−1^) did not show significant changes in Caco‐2 viability, ruling out any toxicity of the developed formulation. Overall, the viability of the CRC cell lines with the modified‐DNPs at different concentrations and functionalization steps suggests that the encapsulated‐DNPs can fulfil their function in the GI for up to 72 h safely.

### 2.4 Cell‐DNP Interaction Studies by Confocal Microscopy

**Figure 5:**
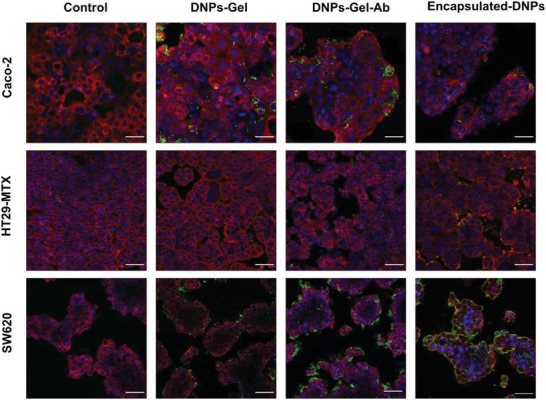
Cell−DNP interactions. Confocal microscopy of Caco‐2, HT29‐MTX, and SW620 cells after incubation with 50 µg of DNPs‐Gel, DNPs‐Gel‐Ab, and encapsulated‐DNPs for 24 h at 37 °C in PBS. CellMask Deep Red (red) was used to stain the cell membranes; DNPs were labeled with Alexa Fluor 488 (green), and nuclei were stained with DAPI (blue). The scale bars are 50 µm. The images were acquired with a Leica SP8 microscope, using a 63× objective.

The chemical composition of the NPs and their surface modifications play a crucial role in the cell‐NP interactions and internalization process. For this reason, the surface of NPs can be tailored to promote specific interactions or mediate the internalization by selected pathways. In choosing the appropriate surface functionalization, the cell membrane composition (i.e., antigen, peptides, and protein expression) must be taken into consideration carefully. Here, to promote the interactions of our formulation with the SW620 cell line specifically, the encapsulated‐DNPs were modified with the anti‐L1‐CAM antibody. We hypothesized that the presence of the antibody on the surface of the NPs could increase the specificity of the interactions with this cell line, rather than with Caco‐2 and HT29‐MTX, which express low levels of L1‐CAM. To evaluate the efficacy of our targeting approach, we studied the interactions of NPs with the three different cell lines by confocal microscopy (**Figure** **5**). The SW620, HT29‐MTX, and Caco‐2 cells were seeded separately and allowed to reach confluency. Then, they were incubated with 50 µg of Alexa Fluor 488‐labeled DNPs‐Gel, DNPs‐Gel‐Ab, and encapsulated‐DNPs in PBS (pH 7.2). At this pH, the HPMC matrix in the encapsulated‐DNPs was dissolved, exposing the inner DNPs‐Gel‐Ab. After 24 h, the cells were extensively washed to remove unspecific interactions, and cell membranes and nuclei were stained. Control cells were incubated with PBS (pH 7.2).

Like many cell lines making epithelial barriers, the Caco‐2 monolayer was characterized by the formation of vacuoles, which make these cells function as gut epithelial cells (Figure 5).^[^ ^53^ ^]^

The DNPs‐Gel were randomly internalized in this cell line, as shown by the heterogeneous distribution of the NPs on the cell monolayer. The cell‐adhesion properties of gelatin covering the DNPs could have promoted the unspecific interactions of the DNPs with the Caco‐2 cells. The DNPs‐Gel and DNPs‐Gel‐Ab showed very similar interactions with these cells, and in both cases, we did not observe any pattern in the cell‐DNP interaction. The random DNP distribution on the cell monolayer suggests that the binding of the DNPs‐Gel and DNPs‐Gel‐Ab was driven by unspecific adsorption on the cell surface. The internalization of the encapsulated‐DNPs in the Caco‐2 cell line was reduced compared to the DNPs‐Gel and DNPs‐Gel‐Ab uptake, confirming that the antibody did not drive the cell‐DNP interaction, even though ca. 20% of the Caco‐2 cells expressed the L1‐CAM (Figure 3). Similar results were observed with the goblet‐like cells HT29‐MTX, which exhibited ca. 10% positivity to the antigen L1‐CAM. Both the DNPs‐Gel and DNPs‐Gel‐Ab showed a few interactions with the HT29‐MTX population. The encapsulated‐DNPs seemed to have been internalized in HT29‐MTX cells more than the DNPs‐Gel and DNPs‐Gel‐Ab, but the result was not comparable to the SW620 cell line overall. The improved internalization of the encapsulated‐DNPs in the epithelial cells can be associated with the reduced size and PDI provided by the encapsulation process. Differently from the epithelial cells HT29‐MTX and Caco‐2, the binding of the encapsulated‐DNPs to the targeted cell line overexpressing the antigen was strongly promoted by the presence of the antibody on the surface of NPs. We observed that the antibody‐labeled formulation interacted better than DNPs‐Gel with the SW620 cells and that the DNPs‐Gel‐Ab were preferentially concentrated at the cell membrane, where the L1‐CAM was expressed. The interactions of the final formulation with the targeted cells appeared highly uniform, as demonstrated by the homogeneous distribution of the encapsulated‐DNPs on the cell monolayer. Therefore, both the good size distribution provided by the microfluidic process and the active targeting approach enhanced the uptake of the encapsulated‐DNPs in the metastatic cell line. The interaction of the developed oral formulation with the SW620, Caco‐2, and HT29‐MTX cells was further compared in Figure S5, which reports the fluorescence signals from the single channels. The green fluorescence represents the labeled‐NPs, and the red and blue channels represent the cell membranes and nuclei, respectively. The amount of the encapsulated‐DNPs detected in the green channel (Figure S5, Supporting Information) was more consistent when NPs were incubated with the metastatic cell line rather than with Caco‐2 or HT29‐MTX cells. The antigen‐antibody binding efficiently enhanced the interactions of the NPs with the cell line expressing high levels of the antigen, as seen for the SW620 cells. On the contrary, its effect was negligible for cells expressing basal levels of L1‐CAM, such as the Caco‐2 and HT29‐MTX cell lines. The obtained results provided qualitative information on the interaction of the encapsulated‐DNPs with the three selected cell lines. However, the detection of cell‐DNP interactions may be negatively impacted by particular features of the cell monolayer, such as the cytoplasmatic vacuolization observed only in Caco‐2 cells (Figure S5, Supporting Information). To further support the findings obtained by confocal microscopy, we measured the uptake of the developed formulation in the cells and quantified the efficacy of the active targeted approach by FACS.

### 2.5 Specificity of the Antibody‐Labeled DNP‐Cell Uptake by FACS

**Figure 6:**
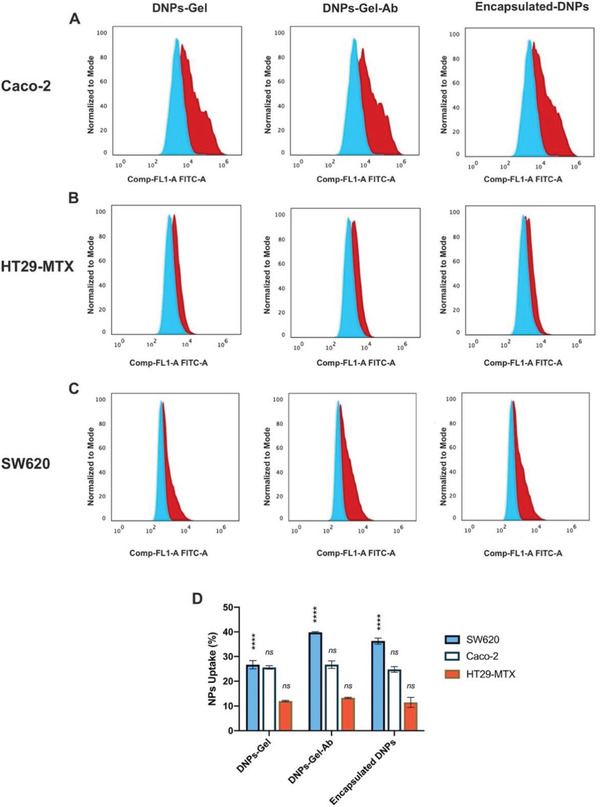
Cell uptake studies. Normalized fluorescence intensity after incubation of A) Caco‐2, B) HT29‐MTX, and C) SW620 cells with the Alexa‐labeled DNPs‐Gel, DNPs‐Gel‐Ab, and encapsulated DNPs for 24 h. Control cells (blue histogram) were incubated with PBS and analyzed to measure cell autofluorescence. After incubation with DNPs, cells (red histogram) were sorted by green fluorescence in the FITC channel. D) Statistical analysis of DNPs’ uptake. The level of significance was set at probabilities of * *p* < 0.05, ** *p* < 0.01, and *** *p* < 0.001. Not statistically significant values are reported as “ns”.

The number of DNPs internalized or firmly bound to the cell membrane was investigated by flow cytometry to quantify the uptake of the encapsulated‐DNPs in the three selected cell lines. According to the quantification of L1‐CAM in the different cell lines (Figure 3) and the interaction studies (Figure 5), the uptake of the encapsulated‐DNPs was expected to be higher in the SW620 than Caco‐2 and HT29‐MTX cells, which express low levels of L1‐CAM. To this aim, metastatic and epithelial cells were seeded separately and allowed to adhere for 24 h. The cells were incubated with the Alexa Fluor^®^488‐labeled DNPs‐Gel, DNPs‐Gel‐Ab, and encapsulated‐DNPs for 24 h in PBS, then the unbound NPs were removed with washings. The cells were detached by trypsin, washed again in a V‐bottom plate and analyzed (**Figure** **6**).

We found that the uptake of both the DNPs‐Gel‐Ab (25.0% ± 0.5%) and encapsulated‐DNPs (26% ± 1%) in Caco‐2 cells was close to the DNPs‐Gel (25% ± 1%) (Figure 6A–D) and mainly unspecific, as also shown by the confocal microscopy analysis (Figure 5). Similarly, the internalization of both the antibody‐labeled and unlabeled DNPs in the HT29‐MTX was comparable to each other, ruling out any specific pattern in the internalization. The average uptake of the DNPs‐Gel, DNPs‐Gel‐Ab, and encapsulated‐DNPs in HT29‐MTX cells was 11% ± 2% of the total amount of NPs incubated with the cells (Figure 6B–D). Therefore, the uptake of the antibody‐labeled DNPs (DNPs‐Gel‐Ab and encapsulated‐DNPs) in the cells expressing basal expression of the antigen (Caco‐2, HT29‐MTX) was mainly unspecific compared to the SW620 cell line. At high antigen concentrations, the functionalization of DNPs‐Gel‐Ab and encapsulated‐DNPs with the antibody anti‐L1‐CAM improved the cell internalization by 13% compared to the untargeted DNPs‐Gel (Figure 6C,D). The uptake of the DNPs‐Gel in the metastatic cells was reported to be 27% ± 2%, which was comparable to the uptake of the same formulation in the Caco‐2 cells. However, the internalization of the DNPs‐Gel‐Ab with the SW620 cells was measured to be 40.0% ± 0.2%, corresponding to an internalization 15% higher than in Caco‐2 and 30% greater than in HT29‐MTX. (Figure 6D).

Overall, the DNPs‐Gel showed a good cell uptake in both Caco‐2 and SW620, confirming the ability of DNPs to penetrate cancer cells and serve as nanocarriers.^[^ ^17^ ^]^ Additionally, the antibody‐labeled formulations (DNPs‐Gel and encapsulated‐DNPs) showed an improved cell uptake in the targeted SW620 cells overexpressing the antigen, due to the antibody‐antigen affinity. The highest NPs’ uptake was observed in the metastatic cell line overexpressing the L1‐CAM (SW620), followed by Caco‐2 and HT29‐MTX, in line with their antigen expression levels (SW620>Caco‐2>HT29‐TMX). This study demonstrates that, even though the DNPs are passively taken up in the CRC cells, the active targeted functionalization moved towards the specific uptake of encapsulated‐DNPs and DNPs‐Gel in the CRC cell line overexpressing the L1‐CAM.

### 2.6 Encapsulated‐DNPs Inhibit the Migration of the Metastatic Cell Line SW620

**Figure 7:**
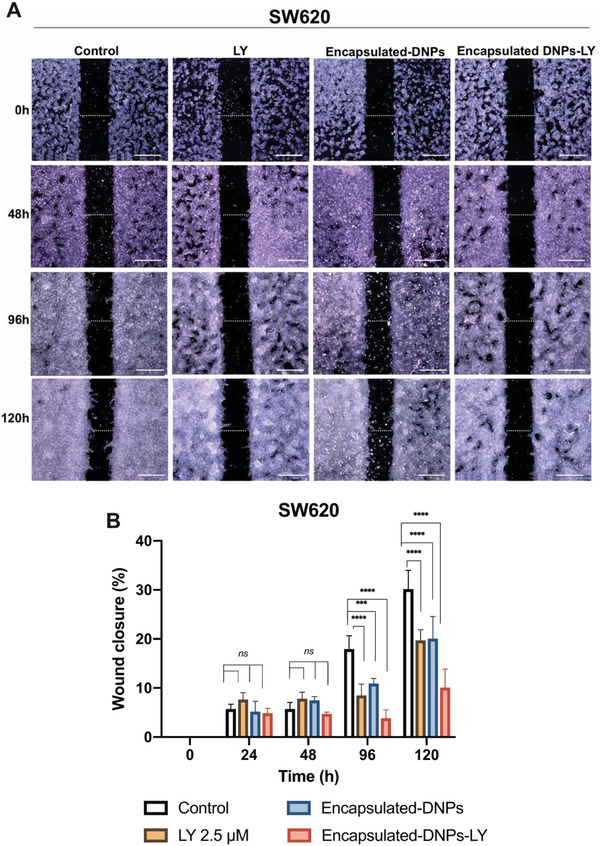
A) Migration assay of SW620 cells. Migration of the cells incubated with 0.5% FBS DMEM (control), LY 2.5 µm, encapsulated‐DNPs 26 µg mL^−1^, and drug‐loaded encapsulated‐DNPs 26 µg mL^−1^ in 0.5% FBS DMEM for 120 h. Images of the wound closure were taken at 0, 24, 48, 96, and 120 h. The size of the closure at time *t* = 0 is 500  µm. The scale bars are 500  µm. Images were acquired with a Leica Microscope and analyzed by the ImageJ software. B) Results are reported as mean ± standard deviation. The level of significance was set at probabilities of * *p* < 0.05, ** *p* < 0.01, and *** *p* < 0.001.

The ability of metastatic cells to migrate and colonize a secondary tumor site is promoted by the upregulation of both the TGF‐*β* pathway and surface expression of adhesion molecules, such as the L1‐CAM.^[^ ^27^ ^]^ The TGF‐*β* pathway promotes the nuclear transcription of pro‐metastatic genes (e.g., Twist, SNAIL, and Vimentin), whereas the L1‐CAM mediates the adaption to the stroma and adhesion to blood capillaries. Hence, the blocking of both the targets (TGF‐*β* and L1‐CAM) can slow down the metastatic cells’ ability to invade and colonize a perivascular site. We investigated whether the release of galunisertib from the encapsulated‐DNPs in the SW620 cells could block the TGF‐*β* pathway, thus reducing the cell migratory capacity. For this purpose, the SW620 cells were seeded in a wound‐healing assay chamber and allowed to reach 90% confluency for 48 h. After that, we studied the migration of the control cells incubated with DMEM supplemented with 0.5% FBS to slow down cell proliferation. To compare the effect of free galunisertib to that of the drug delivered by our NPs, we dispersed either galunisertib (LY) 2.5 µm or drug‐loaded encapsulated DNPs 26 µg mL^−1^ (reported as encapsulated‐DNPs‐LY in **Figure** **7**) in the culture media and studied the cell migration under starvation. The mass of drug‐loaded encapsulated‐DNPs that released an amount of galunisertib equivalent to the free drug was calculated by the drug release studies (see Section 2.2). Then, to evaluate any contribution of the developed NPs on the cell migration, we also investigated the effect of the empty encapsulated‐DNPs (26 µg mL^−1^) on the cells (reported as encapsulated‐DNPs in Figure 7). The cell migration was measured as the percentage of wound closure (Figure 7).

The migration of the untreated cells started only after 48 h and reached 30 ± 3% of wound closure after 120 h in starvation (Figure 7A,B). Cells were kept under starvation in 0.5% FBS DMEM to slow down cell proliferation, which could contribute to wound closure and affect the result of the experiment. Therefore, the migration observed in the control cells (30%) was only due to the capacity of cells to migrate towards the wound area and was not affected by their proliferation. The effect of galunisertib on the cell line was negligible within 48 h and started to be consistent at 96 h when cells began to invade the wound area (Figure 7B). The treatment of the SW620 cells with 2.5 µm of the free drug decreased the wound closure from 30 ± 3% of the control cells to 20 ± 2%, confirming the capacity of galunisertib to downregulate the TGF‐*β* signaling, inhibiting cell migration by 10% compared to the control.

A reduced migration was also observed when SW620 cells were incubated with the empty encapsulated‐DNPs (not loaded with galunisertib), which showed migration of 20% ± 4% after 120 h. The decreased migration of the cells treated with the encapsulated‐DNPs could be due to the presence of the nanocarrier bound on the cell surface and interacting with the antigen L1‐CAM.^[^ ^56^ ^]^ It has been shown that the L1‐CAM has a binding site to several integrins (i.e., *α*5*β*1, *α*v*β*1, and *α*v*β*3) and triggers cell migration through an integrin‐dependent pathway.^[^ ^57^ ^]^ The main integrin binding site was mapped in the sixth immunoglobulin (Ig) domain of the cell adhesion molecule. The inactivation of the integrin binding site on L1‐CAM using antibodies or site‐directed mutagenesis prevents the nuclear translocation of the L1‐CAM signal, reducing cell‐cell adhesion and migration.^[^ ^58^ ^]^ Hence, we suppose that the anchoring of the antibody‐labeled DNPs (encapsulated‐DNPs) to the L1‐CAM on SW620 cells blocked the main integrin binding site, inhibiting their interaction with L1‐CAM and consequent migration. This hypothesis is based on the evidence that L1‐CAM and L1‐CAM‐binding integrins are often expressed by the same cell.^[^ ^59^ ^]^

The inhibition of the migratory properties of SW620 was even stronger when encapsulated‐DNPs were loaded with galunisertib. The cells treated with the drug‐loaded encapsulated‐DNPs showed a migration of 10% ± 3%, which is 11% lower than the migration of cells treated with the free drug, demonstrating an enhanced effect of the delivered galunisertib (Figure 7A,B). The improved effect of galunisertib delivered by DNPs in CRC was already validated in previous works, in which we reported a 3‐fold increase of the gene downregulation by the delivered drug compared to the free molecule.^[^ ^13^ ^]^ Here, the increased therapeutic effect was provided by both the drug local release and blocking of L1‐CAM. The binding of the NPs to the L1‐CAM inhibited the interactions between integrins and the surface antigen. Therefore, the active‐targeted approach not only improved the uptake of NPs in the targeted cell line but also inhibited the EMT process mediated by the engagement of integrins by the surface antigen. Overall, the synergistic effect of the drug and L1‐CAM blocking produced a therapeutic outcome that was greater than the inhibition of migration induced by the free form of galunisertib.

## 3 Conclusion

Here, we reported the development of galunisertib‐loaded gelatin‐covered diatomite NPs modified with an antibody and further entrapped in HPMC by microfluidic nanoprecipitation. This unique approach allowed the encapsulation of the active‐targeted NPs with great efficiency, obtaining a formulation with good size distribution and gastro‐resistant features for local drug release. The enteric polymer protected the gelatin coverage on NPs from enzymatic degradation in the stomach and triggered the release of galunisertib at the small intestine and colon pH. The developed formulation showed high biocompatibility with three colon cancer cell lines classifiable as either metastatic (SW620) or epithelial (Caco‐2, HT29‐MTX). The higher antigen levels expressed by the SW620 cells made them the ideal target of the developed NPs. The gastro‐resistant formulation was designed to ease the binding of the anti‐L1‐CAM antibodies, exposed on the NPs’ surface, to SW620 cell membrane antigens after the dissolution of the enteric matrix in the colon. The internalization and uptake studies demonstrated that the antigen‐antibody interactions improved the uptake of NPs in the cell line overexpressing the antigen. In contrast, this approach had negligible effects on the cells expressing basal levels of the L1‐CAM antigen (Caco‐2 and HT29‐MTX). Finally, we demonstrated that the delivered galunisertib was capable of slowing down the migration of the SW620 cells by blocking the TGF‐*β* pathway with higher efficiency than the free drug. The obtained results suggest that the effect of the delivered drug on the cells was enhanced by the inhibition of the interactions between L1‐CAM and integrins. Overall, the increased galunisertib concentration at the target site provided by the enteric matrix, and the L1‐CAM inhibition by the active‐targeted NPs could lead to enhanced therapeutic outcomes for CRC treatment.

## 4 Experimental Section

### Materials

The materials and reagents used in the following studies are described in detail in the Supporting Information.

### Fabrication of Galunisertib‐Loaded Gelatin‐Modified DNPs (DNPs‐Gel)

DNPs were produced by sonication and purification of the diatomite powder, and amino‐silanized as reported elsewhere.^[^ ^12^, ^13^ ^]^ The detailed protocol of DNPs production and amino‐silanization is reported in the Supporting Information section. Galunisertib was loaded in amino‐modified DNPs by an immersion method. First, 0.1 mg of amino‐modified DNPs were dispersed in a 0.125 mg mL^−1^ galunisertib solution and stirred for 2 h (400 rpm). Then, the dispersion was centrifuged, the supernatant was removed and the drug‐loaded DNPs were immersed in MES solution with gelatin 5 mg mL^−1^ and stirred for 2 h. A mixture of EDC (final concentration 3.8 µm) and NHS (final concentration 1.7 µm) was added to the dispersion to crosslink the gelatin via the formation of an intramolecular peptide bond. After 2 h, DNPs‐Gel were centrifuged (13 200 rpm), washed with H_2_O three times, and collected.

### Binding of the Anti‐L1‐CAM Antibody to the Gelatin Coating of DNPs‐Gel

For the binding of the antibody, the DNPs‐Gel were first functionalized with a recombinant Pr‐A to promote the anchoring of the antibody to DNPs via the Fc rather than the Fab fragment. For this reason, 0.1 mg of the DNPs‐Gel were suspended in PBS 1 × pH 7.2 solution of Pr‐A 0.1 mg mL^−1^ with 2 µm EDC and 0.8 µm NHS for 2 h (400 rpm). Then, the suspension was centrifuged, the supernatant was removed and the NPs were washed three times with PBS. Pr‐A‐modified DNPs‐Gel were incubated with a PBS solution of the anti‐L1‐CAM antibody 0.8 µg mL^−1^ for 2 h. Finally, the antibody‐labeled DNPs‐Gel (DNP‐Gel‐Ab) were washed with H_2_O, centrifuged, and collected for encapsulation.

### Production of Encapsulated‐DNPs via Glass Capillary‐Based Microfluidics

As reported elsewhere, the drug‐loaded DNPs‐Gel‐Ab were encapsulated into HPMC using the microfluidics glass capillary technique.^[^ ^23^, ^30^ ^]^ The fabrication of the microfluidic chip consisting of an inner and outer capillary is reported in the Supporting Information section. HPMC is a polymer commonly used for enteric coatings soluble at alkaline pH with an opening pH range between 5.5 and 6.5.^[^ ^60^ ^]^ The inner phase was injected through the inner capillary and consisted of drug‐loaded DNPs‐Gel‐Ab (1 mg mL^−1^) dispersed in ethanol: water solution (50:50) of HPMC (5 mg mL^−1^). The outer solution phase was injected in the outer capillary and consisted of a water solution of PVA 0.5% at pH 6.7. The inner solution was injected at 1 mL h^−1^, and the outer solution at 30 mL h^−1^ (1:30 ratio). Based on different flow rate ratios, we obtained formulations with varying size, *ζ*‐potential, and polydispersity (Table S1, Supporting Information). The flow rate ratio 1:30 enabled us to obtain encapsulated‐DNPs with the desired size, PDI, and morphology, as confirmed by TEM analysis. The encapsulated‐DNPs were collected from the end of the outer capillary under mild stirring (400 rpm), centrifuged (13 200 rpm), and washed with H_2_O to remove the excess reagents.

### Physicochemical Characterization

Hydrodynamic diameter (Z‐average), polydispersity index (PDI), and zeta (*ζ*)‐potential of the NPs were measured using a Zetasizer ZS Nano instrument (Malvern Instruments Ltd., UK). The functionalization steps (gelatin coating, antibody labeling, and encapsulation in the HPMC polymer) were evaluated using a Bruker VERTEX 70 series FTIR spectrometer (Bruker Optics, Germany) with a horizontal ATR sampling accessory (MIRacle, Pike Technology, USA) with a resolution of 4 cm^−1^. The analysis was performed with dried samples (0.5–1 mg mL^−1^) at RT.

The morphology of bare DNPs, DNPs‐Gel‐Ab, and encapsulated‐DNPs was evaluated by TEM, using a TecnaiTM F12 microscope (FEI Company, USA). For this purpose, NPs were dispersed in H_2_O at the final concentration of 0.1 mg mL^−1^, pipetted on copper‐coated grids, left to dry at RT, and analyzed. The encapsulation of the DNPs‐Gel‐Ab in HPMC was assessed using an Oxford INCA 350 EDX spectrometer connected with a field emission scanning electron microscope (FESEM; Hitachi S‐4800, Japan). The measurement points were selected from areas imaged with the bright field TE detector of the FESEM.

### Galunisertib In Vitro Loading and Release Studies

The drug loading capacity (LC) of the encapsulated‐DNPs was determined by immersing NPs (0.1 mg) in 1 mL of ethanol 100% for 4 h to dissolve the polymer matrix under stirring (400 rpm). The dispersion was centrifuged (13 200 rpm), the supernatant collected, and the DNPs‐Gel‐Ab obtained by the HPMC dissolution were dispersed in 1 mL of PBS pH 7.2. Trypsin (0.06 mg) was added to this solution to degrade the gelatin matrix and favor the release of galunisertib, as reported previously.^[^ ^12^ ^]^ After 2 h, the encapsulated‐DNPs were centrifuged (13 200 rpm), and the released drug in the supernatants was quantified by RP‐HPLC using a Discovery C18 Column (Merck, DE) as stationary phase (5 µm particle size, 150 × 4.6 mm). Mobile phase A comprised TFA 0.02% v/v in H_2_O, whereas mobile phase B was TFA 0.02% v/v in acetonitrile (ACN). The flow rate and wavelength were set at 1 mL min^−1^ and 254 nm, respectively. The amount of loaded/released drug was quantified using an external calibration method and the LC was determined using Equation (1):

(1) $$\mathrm{LC}\;\left(\%\right)=\frac{\mathrm{amount}\;\mathrm{of}\;\mathrm{released}\;\mathrm{galunisertib}\;(\mathrm{mg})}{\mathrm{amount}\;\mathrm{of}\;\mathrm{Encapsulated}\;\mathrm{DNPs}\left(\mathrm{mg}\right)}\times 100$$

For the in vitro drug release, the encapsulated‐DNPs were dispersed in buffers mimicking the gastrointestinal (GI) tract (stomach, intestine, and colon), such as the SGF pH 1.6 and FaSSIF, pH 5.5 and 8.0. The SGF solution consisted of 0.2% w/v sodium chloride (NaCl) and 0.7% v/v HCl, to which pepsin (final concentration 1 mg mL^−1^) was added to mimic the presence of digestive enzymes. The FaSSIF solutions consisted of 106 mm NaCl, monobasic sodium phosphate, 8.7 mm sodium hydroxide, 3 mm sodium taurocholate, and 0.75 mm lecithin. The pH of FaSSIF solutions was adjusted to 5.5 and 8.0, and trypsin (final concentration 0.06 mg mL^−1^) was added to mimic the intestine and colon composition. Encapsulated‐DNPs were dispersed in SGF‐pepsin^+^ for 2 h at 37 °C and under stirring (400 rpm). Then, the dispersion was centrifuged (13 200 rpm), the supernatants were collected at different time points (5, 15, 30, 60, and 120 min), and the NPs resuspended in pre‐warmed fresh buffer. After 2 h, the NPs were removed from the SGF solution by centrifugation for 5 min at 13 200 rpm and dispersed in FaSSIF‐trypsin^+^ pH 5.5 for 4 h at 400 rpm. The supernatants were collected at different time points (5, 15, 30, 60, 120, 180, and 240 min) by centrifugation (13 200 rpm), and analyzed. Finally, encapsulated‐DNPs were dispersed in FaSSIF‐trypsin^+^ pH 8.0 for 2 h at 400 rpm. The NPs were centrifuged at different points (5, 15, 30, 60, and 120 min) to collect the supernatant and calculate the released drug by RP‐HPLC. The cumulative percentage release was calculated by using Equations (2) and (3):

(2) $$\mathrm{Released}\;\mathrm{drug}\;\left(\%\right)=\frac{\mathrm{released}\;\mathrm{drug}\;\mathrm{at}\;\mathrm{time}\;t}{\mathrm{total}\;\mathrm{amount}\;\mathrm{of}\;\mathrm{drug}\;\mathrm{loaded}}\times 100$$

(3) $$\mathrm{Cumulative}\;\mathrm{release}\left(\%\right)=P\left(t-1\right)+Pt$$

where ***P**t* is the percentage of the released drug at time *t* and P(*t* − 1) is the percentage of the drug quantified at the previous time.

### Dissolution Behavior of Encapsulated‐DNPs in Buffers Mimicking the GI Tract

To evaluate the dissolution behavior of the encapsulated‐DNPs in the GI tract, NPs were dispersed in the buffer solutions SGF and FaSSIF (pH 1.6, 5.5, and 8.0) supplemented with enzymes at 37 °C and under magnetic stirring. Samples were collected at different time points (5, 30, 60, and 120 min), washed, and placed on copper‐coated grids. The encapsulated‐DNPs were left to dry over the grid at RT and then observed by TEM microscopy.

### Cell Lines and Culture Conditions

Human colon adenocarcinoma Caco‐2 cells were obtained from the American Type Culture Collection (ATCC, USA). Human goblet‐like HT29‐MTX was kindly provided by Dr T. Lesuffleur (INSERM U178, Villejuif, France). Caco‐2 (passages #30–40) and HT29‐MTX (passages #30‐37) cells were grown separately in tissue culture flasks (Corning Inc., USA) with high‐glucose Dulbecco's Modified Eagle's Medium (DMEM) supplemented with 10% v/v heat‐inactivated fetal bovine serum (FBS), 1% v/v L‐glutamine, 1% v/v non‐essential amino acids (NEEA), 1% v/v and antibiotic–antimitotic mixture (final concentration of Penicillin and Streptomycin (PEST) 100 IU mL^−1^). SW620 (passages #24‐32) were purchased from Cell Line Service GmbH (CLS, DE) and cultured in tissue flasks with high glucose DMEM supplemented with 10% v/v FBS, 1% v/v L‐glutamine, and 1% v/v PEST. Cells were kept in the incubator (16 BB gas, Heraeus Instruments GmbH, Germany) at 37 °C and 5% CO_2_ in a water‐saturated atmosphere. For each cell line, the culture medium was replaced every other day and sub‐culturing was performed at 80% confluency for all the cell lines using trypsin‐PBS‐EDTA.

### Cell Viability Studies

The cell viability of Caco‐2, HT29‐MTX, and SW620 was evaluated with the CellTiter‐Glo Luminescent Cell Viability Assay, measuring the amount of ATP produced by living cells. Cells (5  × 10^5^ cells mL^−1^) were seeded separately into 96‐well plates and allowed to attach for 24 h. Then, the cell culture medium was removed, and DNPs, DNPs‐Gel, DNPs‐Gel‐Ab, and encapsulated‐DNPs were incubated with Caco‐2, HT29‐MTX, and SW620 cells at different concentrations (12, 25, 50, and 100 µg mL^−1^) for 6 and 24 h at 37 °C, 5% CO_2_. After incubation, cells were washed twice with HBSS and HEPES, and then incubated with 0.1 mL of CellTiter‐Glo (prepared in HBSS–HEPES buffer ratio 1:1). Positive and negative controls were obtained by incubating cells with Triton X‐100 1% and HBSS–HEPES buffer, respectively. The plates were shaken for 5 min, and the luminescence was measured using a Varioskan Microplate Reader (ThermoFisher Scientific, USA). The experiments were carried out in triplicates (*n* ≥ 3).

### Quantification of the Expression Levels of the Cell Surface Antigen L1‐CAM

For this study, 3 × 10^5^ Caco‐2, HT29‐ MTX, and SW620 cells were detached with trypsin from the flasks, washed in PBS, and seeded separately in 96‐well V‐Bottom plates for fluorescence‐assisted cell sorting (FACS) analysis. This technique can be used to complement the NP‐cell interaction studies obtained by confocal microscopy.^[^ ^61^ ^]^ The cells were incubated with the anti‐L1‐CAM primary antibody solution 6 µg mL^−1^ for 30 min at 4 °C (volume of staining 0.1 mL, 50:50 cells: antibody). Then, the cells were centrifuged, washed twice with PBS and incubated with the secondary antibody m‐IgGK BP‐CFL‐647 2 µg mL^−1^ for 30 min, RT, in dark conditions. Control cells were incubated with only the secondary antibody to measure the binding unspecificity. Cells were sorted by fluorescence in the APC channel with an Accuri C6 Plus Flow Cytometer (BD Biosciences). For each condition, a minimum of 2 × 10^4^ events were measured. Flow cytometry data were analyzed using FlowJo.

### Cell‐DNP Interaction Studies

To study the interaction of NPs with Caco‐2, HT29‐MTX and SW620 cells, 2  × 10^5^ cells mL^−1^ was seeded in Lab‐Tek eight‐chamber slides (ThermoFisher Scientific) separately and allowed to attach for 24 h in humidified atmosphere. Then, the medium was discarded and cells were incubated with 0.05 mg of Alexa Fluor 488‐labeled DNPs‐Gel, DNPs‐Gel‐Ab, and encapsulated‐DNPs in DMEM for 24 h. The fluorophore labeling was performed according to the protocol described elsewhere.^[^ ^13^ ^]^ After reaching confluency, cells were washed twice with HBSS‐HEPES, fixed by paraformaldehyde (PFA) 1% v/v, and nuclei and membrane were stained with 6‐diamidino‐2‐phenylindole dihydrochloride (DAPI) and CellMask Deep Red, respectively. Finally, cells were washed and suspended in fresh PBS and analyzed by Leica SP5 II HCS‐A confocal microscope (Leica Microsystems, Wentzler, Germany). Images were analyzed using Fiji software.

### Cell Uptake of DNPs Measured by Flow Cytometry (FACS)

Caco‐2, HT29‐MTX, and SW620 cells were seeded separately in a 24‐well plate at a density of 1  × 10^5^ cells per well and incubated for 24 h at 37 °C and 5% CO_2_. Cells were washed once with PBS pH 7.2 and incubated with Alexa Fluor 488‐labeled DNPs‐Gel, DNPs‐Gel‐Ab, and encapsulated‐DNPs 0.05 mg mL^−1^ in DMEM for 24 h in a humidified atmosphere. Cells were washed twice with PBS to remove the non‐internalized NPs before detachment and centrifuged at 13 200 rpm for 4 min. Then, cells were suspended in PBS in a 96‐well V‐Bottom plate and analyzed by FACS (Accuri C6 Plus Flow Cytometer, BD Biosciences) 0.2 × 10^5^ cells were measured and sorted by fluorescence in the FITC channel for each condition.

### Migration Assay

For the investigation of Caco‐2, HT29‐ MTX, and SW620 migration, cells were seeded separately into culture‐inserts 2 well in *µ*‐dishes 35 mm (IBIDI, IT) at a density of 8  × 10^4^ cells per well in DMEM supplemented with FBS 10%. Cells were left to adhere and reach 95% confluency for 48 h at 37 °C and humified atmosphere. After 48 h, the insert was removed, and the cells were washed and allowed to migrate in DMEM 0.5% FBS (control) under starvation. The efficacy of both the free and delivered drug was investigated by supplementing DMEM 0.5% FBS with either free galunisertib (LY) 2.5 µm or encapsulated drug‐loaded DNPs (Encapsulated DNPs‐LY, 26 µg mL^−1^) for 24 h, and quantifying the cell migration over time. The concentration of the encapsulated‐DNPs‐LY releasing an equivalent amount of drug (2.5 µm) was calculated by the drug release studies (Galunisertib In Vitro Loading and Release Studies, Experimental Section). We also investigated the effect of the empty encapsulated‐DNPs (26 µg mL^−1^) to assess if any of the components of the formulation affected cell migration. After 24 h, the cells were washed twice with DMEM to remove the drug and NPs dispersed in the media, and fresh DMEM 0.5% FBS was added to the plate to keep the cells under starvation. The culture medium was replaced every day. The pictures were acquired with an inverted microscope (Leica Microsystem, GmbH) and analysis was performed using Fiji software.

### Statistical Analysis

All experiments were performed at least in triplicates, and the measured values are expressed as mean ± standard deviation (s.d.). Results were evaluated by means of two‐way analysis of variance (ANOVA) with the Bonferroni test, and levels of significance are set at probabilities of * *p* < 0.05, ** *p* < 0.01, *** *p* < 0.001, **** *p* < 0.0001.

## Conflict of Interest

The authors declare no conflict of interest.

## Supporting information

Supporting Information

## Data Availability

The data that support the findings of this study are available from the corresponding author upon reasonable request.

## References

[1] H. Cheng , S. Huang , G. Huang , J. Enzyme Inhib. Med. Chem. 2019, 34, 1590. 31581863 10.1080/14756366.2019.1655406 PMC6781185

[2] A. Tiwari , S. Saraf , A. Verma , P. K. Panda , S. K. Jain , World J. Gastroenterol. 2018, 24, 4428. 30357011 10.3748/wjg.v24.i39.4428 PMC6196338

[3] D. R. Rivas , M. V. C. Dela Cerna , C. N. Smith , S. Sampathi , B. G. Patty , D. Lee , J. S. Blackburn , Sci. Rep. 2021, 11, 10302. 33986418 10.1038/s41598-021-89668-5 PMC8119466

[4] B. Rani , A. Malfettone , F. Dituri , J. Soukupova , L. Lupo , S. Mancarella , I. Fabregat , G. Giannelli , Cell Death Dis. 2018, 9, 373. 29515105 10.1038/s41419-018-0384-5 PMC5841307

[5] E. Dekker , P. J. Tanis , J. L. A. Vleugels , P. M. Kasi , M. B. Wallace , Lancet 2019, 394, 1467. 31631858 10.1016/S0140-6736(19)32319-0

[6] F. Di Nicolantonio , P. P. Vitiello , S. Marsoni , S. Siena , J. Tabernero , L. Trusolino , R. Bernards , A. Bardelli , Nat. Rev. Clin. Oncol. 2021, 18, 506. 33864051 10.1038/s41571-021-00495-z

[7] Y. Xu , B. Pasche , Hum. Mol. Genet. 2007, 16, R14. 17613544 10.1093/hmg/ddl486 PMC2637552

[8] K. C. Cassidy , I. Gueorguieva , C. Miles , J. Rehmel , P. Yi , W. J. Ehlhardt , Xenobiotica 2018, 48, 382. 28436712 10.1080/00498254.2017.1323137

[9] Y. Dang , J. Guan , Smart Mater. Med. 2020, 1, 10. 34553138 10.1016/j.smaim.2020.04.001 PMC8455119

[10] M. J. Mitchell , M. M. Billingsley , R. M. Haley , M. E. Wechsler , N. A. Peppas , R. Langer , Nat. Rev. Drug Discovery 2021, 20, 101. 33277608 10.1038/s41573-020-0090-8 PMC7717100

[11] L. L. van Leeuwen , H. G. Leuvenink , B. M. Kessler , P. Olinga , M. J. Ruigrok , bioRxiv 2022, 10.1101/2022.03.22.485255 37596999

[12] C. Tramontano , B. Miranda , G. Chianese , L. De Stefano , C. Forestiere , M. Pirozzi , I. Rea , Iran J. Med. Sci. 2021, 22, 10755. 10.3390/ijms221910755 PMC8509241 34639096

[13] S. Managò , C. Tramontano , D. Delle Cave , G. Chianese , G. Zito , L. De Stefano , M. Terracciano , E. Lonardo , A. C. De Luca , I. Rea , Small 2021, 17, 2101711. 10.1002/smll.202101711 34302422

[14] M. Terracciano , M.‐A. Shahbazi , A. Correia , I. Rea , A. Lamberti , L. De Stefano , H. A. Santos , Nanoscale 2015, 7, 20063. 26568517 10.1039/c5nr05173h

[15] C. Tramontano , G. Chianese , M. Terracciano , L. de Stefano , I. Rea , Appl. Sci. 2020, 10, 6811.

[16] J. Delasoie , F. Zobi , Pharmaceutics 2019, 11, 537. 31618958 10.3390/pharmaceutics11100537 PMC6835591

[17] I. Rea , N. M. Martucci , L. D. Stefano , I. Ruggiero , M. Terracciano , P. Dardano , N. Migliaccio , P. Arcari , R. Taté , I. Rendina , A. Lamberti , Biochim. Biophys. Acta, Gen. Subj. 2014, 1840, 3393. 10.1016/j.bbagen.2014.09.009 25224732

[18] S. Managò , N. Migliaccio , M. Terracciano , M. Napolitano , N. M. Martucci , L. De Stefano , I. Rendina , A. C. De Luca , A. Lamberti , I. Rea , J. Biophotonics 2018, 11, 201700207. 10.1002/jbio.201700207 29144609

[19] C. Tramontano , L. De Stefano , M. Terracciano , G. Chianese , I. Rea , in AlgalBiotechnology (Eds: A. Ahmad , F. Banat , H. Taher ), Elsevier, Amsterdam, 2022, pp. 427–446.

[20] M. Terracciano , F. Fontana , A. P. Falanga , S. D'Errico , G. Torrieri , F. Greco , C. Tramontano , I. Rea , G. Piccialli , L. De Stefano , G. Oliviero , H. A. Santos , N. Borbone , Small 2022, 18, 2204732. 10.1002/smll.202204732 36089668

[21] Z. Liu , F. Fontana , A. Python , J. T. Hirvonen , H. A. Santos , Small 2020, 16, 2070048. 10.1002/smll.201904673 31702878

[22] S. Siavashy , M. Soltani , M. Ahmadi , B. Landi , H. Mehmanparast , F. Ghorbani‐Bidkorbeh , Adv. Mater. Technol. 2022, 7, 2101615.

[23] W. Li , D. Liu , H. Zhang , A. Correia , E. Mäkilä , J. Salonen , J. Hirvonen , H. A. Santos , Acta Biomater. 2017, 48, 238. 27815166 10.1016/j.actbio.2016.10.042

[24] S. Jung , J. Lee , J. Lim , J. Suh , T. Kim , J. Ahn , W. J. Kim , Y. Kim , Adv. Healthcare Mater. 2020, 9, 2001633. 10.1002/adhm.202001633 PMC7677199 33073526

[25] F. Tian , L. Cai , C. Liu , J. Sun , Lab Chip 2022, 22, 512. 35048096 10.1039/d1lc00812a

[26] C. De Pascalis , S. Etienne‐Manneville , Mol. Biol. Cell 2017, 28, 1833. 28684609 10.1091/mbc.E17-03-0134 PMC5541834

[27] H. Schäfer , B. Struck , E.‐M. Feldmann , F. Bergmann , E. Grage‐Griebenow , C. Geismann , S. Ehlers , P. Altevogt , S. Sebens , Oncogene 2013, 32, 180. 22349829 10.1038/onc.2012.44

[28] D. D. Cave , X. Hernando‐Momblona , M. Sevillano , G. Minchiotti , E. Lonardo , Theranostics 2021, 11, 5686. 33897875 10.7150/thno.54027 PMC8058729

[29] F. Araújo , N. Shrestha , M.‐A. Shahbazi , D. Liu , B. Herranz‐Blanco , E. M. Mäkilä , J. J. Salonen , J. T. Hirvonen , P. L. Granja , B. Sarmento , H. A. Santos , ACS Nano 2015, 9, 8291. 26235314 10.1021/acsnano.5b02762

[30] J. P. Martins , D. Liu , F. Fontana , M. P. A. Ferreira , A. Correia , S. Valentino , M. Kemell , K. Moslova , E. Mäkilä , J. Salonen , J. Hirvonen , B. Sarmento , H. A. Santos , ACS Appl. Mater. Interfaces 2018, 10, 44354. 30525379 10.1021/acsami.8b20821

[31] A. N. Ilinskaya , M. A. Dobrovolskaia , Nanomedicine 2013, 8, 969. 23730696 10.2217/nnm.13.49 PMC3939602

[32] D. Chenthamara , S. Subramaniam , S. G. Ramakrishnan , S. Krishnaswamy , M. M. Essa , F.‐H. Lin , M. W. Qoronfleh , Biomater. Res. 2019, 23, 20. 31832232 10.1186/s40824-019-0166-x PMC6869321

[33] P. Jani , G. W. Halbert , J. Langridge , A. T. Florence , J. Pharm. Pharmacol. 1989, 41, 809. 2576440 10.1111/j.2042-7158.1989.tb06377.x

[34] W. Yu , R. Liu , Y. Zhou , H. Gao , ACS Cent. Sci. 2020, 6, 100. 32123729 10.1021/acscentsci.9b01139 PMC7047275

[35] H. Luo , L. Kong , F. Zhang , C. Huang , J. Chen , H. Zhang , H. Yu , S. Zheng , H. Xu , Y. Zhang , L. Deng , G. Chen , H. A. Santos , W. Cui , Adv. Funct. Mater. 2021, 31, 2101262.

[36] C. Cha , J. Oh , K. Kim , Y. Qiu , M. Joh , S. R. Shin , X. Wang , G. Camci‐Unal , K. Wan , R. Liao , A. Khademhosseini , Biomacromolecules 2014, 15, 283. 24344625 10.1021/bm401533y PMC3922064

[37] S. Van Vlierberghe , J. Mater. Sci. 2016, 51, 4349.

[38] M. Chen , G. Bolognesi , G. T. Vladisavljević , Molecules 2021, 26, 3752. 34202959 10.3390/molecules26123752 PMC8234156

[39] W. Choe , T. A. Durgannavar , S. J. Chung , Materials (Basel) 2016, 9, 994. 28774114 10.3390/ma9120994 PMC5456964

[40] M. M. Al‐Tabakha , J. Pharm. Pharm. Sci. 2010, 13, 428. 21092714 10.18433/j3k881

[41] V. Balasubramanian , A. Domanskyi , J.‐M. Renko , M. Sarparanta , C.‐F. Wang , A. Correia , E. Mäkilä , O. S. Alanen , J. Salonen , A. J. Airaksinen , R. Tuominen , J. Hirvonen , M. Airavaara , H. A. Santos , Biomaterials 2020, 227, 119556. 31670035 10.1016/j.biomaterials.2019.119556

[42] J. F. Reinus , D. Simon , Gastrointestinal anatomy and physiology: The essentials, Wiley‐Blackwell, Hoboken, NJ 2014.

[43] A. J. Coupe , S. S. Davis , I. R. Wilding , Pharm. Res. 1991, 8, 360. 2052525 10.1023/a:1015849700421

[44] N. A. Metheny , B. J. Stewart , L. Smith , Hua Yan , M. Diebold , R. E. Clouse , JPEN, J. Parenter. Enteral Nutr. 1997, 21, 279. 9323690 10.1177/0148607197021005279

[45] S. Buhmann , C. Kirchhoff , R. Ladurner , T. Mussack , M. F. Reiser , A. Lienemann , Eur. Radiol. 2007, 17, 669. 17036156 10.1007/s00330-006-0414-z

[46] S. Shen , Y. Wu , Y. Liu , D. Wu , Int. J. Nanomed. 2017, 12, 4085. 10.2147/IJN.S132780 PMC5459982 28615938

[47] S. Tiburcius , K. Krishnan , L. Jose , V. Patel , A. Ghosh , C. I. Sathish , J. Weidenhofer , J.‐H. Yang , N. M. Verrills , A. Karakoti , A. Vinu , Nanoscale 2022, 14, 6830. 35441642 10.1039/d2nr00783e

[48] C. G. Bavnhøj , M. M. Knopp , C. M. Madsen , K. Löbmann , Int. J. Pharm.: X 2019, 1, 100008. 31517273 10.1016/j.ijpx.2019.100008 PMC6733371

[49] A. Courts , Biochem. J. 1955, 59, 382. 14363105 10.1042/bj0590382 PMC1216254

[50] P. Altevogt , A. Ben‐Ze'ev , N. Gavert , U. Schumacher , H. Schäfer , S. Sebens , Int. J. Cancer 2020, 147, 3292. 32588424 10.1002/ijc.33177

[51] R. E. Hewitt , A. McMarlin , D. Kleiner , R. Wersto , P. Martin , M. Tsoskas , G. W. H. Stamp , W. G. Stetler‐Stevenson , J. Pathol. Clin. Res. 2000, 192, 446. 10.1002/1096-9896(2000)9999:9999<::AID-PATH775>3.0.CO;2-K 11113861

[52] M. Hekmati , Y. Ben‐Shaul , S. Polak‐Charcon , Cell Differ. Dev. 1990, 31, 207. 2271997 10.1016/0922-3371(90)90133-h

[53] T. Lea , in The Impact of Food Bioactives on Health: In Vitro and Ex Vivo Models (Eds: K. Verhoeckx , P. Cotter , I. López‐Expósito , C. Kleiveland , T. Lea , A. Mackie , T. Requena , D. Swiatecka , H. Wichers ), Springer International Publishing, Cham 2015, pp. 103–111. 29787039

[54] C. Pontier , J. Pachot , R. Botham , B. Lenfant , P. Arnaud , J. Pharm. Sci. 2001, 90, 1608. 11745719 10.1002/jps.1111

[55] S. Hua , Front. Pharmacol. 2020, 11, 524. 32425781 10.3389/fphar.2020.00524 PMC7212533

[56] M. Ruppert , S. Aigner , M. Hubbe , H. Yagita , P. Altevogt , J. Cell Biol. 1995, 131, 1881. 8557754 10.1083/jcb.131.6.1881 PMC2120661

[57] S. Silletti , M. Yebra , B. Perez , V. Cirulli , M. McMahon , A. M. P. Montgomery , J. Biol. Chem. 2004, 279, 28880. 15128735 10.1074/jbc.M404075200

[58] H. Kiefel , S. Bondong , J. Hazin , J. Ridinger , U. Schirmer , S. Riedle , P. Altevogt , Cell Adh. Migr. 2012, 6, 374. 22796939 10.4161/cam.20832 PMC3478260

[59] D. Gast , S. Riedle , H. Kiefel , S. S. Müerköster , H. Schäfer , M. K. E. Schäfer , P. Altevogt , Exp. Cell Res. 2008, 314, 2411. 18555990 10.1016/j.yexcr.2008.04.004

[60] D. T. Friesen , R. Shanker , M. Crew , D. T. Smithey , W. J. Curatolo , J. A. S. Nightingale , Mol. Pharmaceutics 2008, 5, 1003. 10.1021/mp8000793 19040386

[61] L. Woythe , P. Madhikar , N. Feiner‐Gracia , C. Storm , L. Albertazzi , ACS Nano 2022, 16, 3785. 35274534 10.1021/acsnano.1c08277 PMC8945370

