# Supplementary material for: Microfluidic‐Assisted Production of Gastro‐Resistant Active‐Targeted Diatomite Nanoparticles for the Local Release of Galunisertib in Metastatic Colorectal Cancer Cells
Source: Adv Healthc Mater. 2022 Dec 11;12(6):2202672. doi: 10.1002/adhm.202202672 (PMC11468342; doi:10.1002/adhm.202202672)
Supplement: Supplementary file 1 — Supporting Information [file ADHM-12-2202672-s001.pdf]

ADVANCED HEALTHCARE MATERIALS

Supporting Information

for *Adv. Healthcare Mater.*, DOI 10.1002/adhm.202202672

Microfluidic-Assisted Production of Gastro-Resistant Active-Targeted Diatomite Nanoparticles for the Local Release of Galunisertib in Metastatic Colorectal Cancer Cells

Chiara Tramontano, João Pedro Martins, Luca De Stefano, Marianna Kemell, Alexandra Correia, Monica Terracciano, Nicola Borbone, Ilaria Rea and Hélder A. Santos**

Supporting Information

Microfluidic-assisted production of gastro-resistant active-targeted diatomite nanoparticles for the local release of galunisertib in metastatic colorectal cancer cells

Chiara Tramontano, João Pedro Martins, Luca De Stefano, Marianna Kemell, Alexandra Correia, Monica Terracciano, Nicola Borbone, Ilaria Rea and Hélder A. Santos**

C. Tramontano, L. De Stefano, I. Rea

Institute of Applied Sciences and Intelligent Systems, Unit of Naples, National Research Council, Naples, 80131, Italy
E-mail: ilaria.rea@na.isasi.cnr.it

J.P. Martins, Alexandra Correia, H.A. Santos
Drug Research Program, Division of Pharmaceutical Chemistry and Technology, Faculty of Pharmacy, University of Helsinki, FI-00014, Helsinki, Finland

M. Kemell

Department of Chemistry, University of Helsinki, FI-00014, Helsinki, Finland

M. Terracciano, N. Borbone, C. Tramontano

Department of Pharmacy, University of Naples Federico II, 80131, Naples, Italy

H.A. Santos

Department of Biomedical Engineering, University Medical Center Groningen, University of Groningen, 9713 AV Groningen, The Netherlands

E-mail: h.a.santos@umcg.nl

H.A. Santos

W.J. Kolff Institute for Biomedical Engineering and Materials Science, University Medical Center Groningen, University of Groningen, 9713 AV, Groningen, The Netherlands

1. Materials and Methods

Diatomite powder was kindly provided by DEREF Spa (IT); 1-ethyl-3-[3-dimethylaminopropyl]carbodiimide-hydrochloride (EDC), N-hydroxysuccinimide (NHS), 3-aminopropyltriethoxysilane (APTES), type-B gelatin, 2-(N-Morpholino) ethane sulfonic acid hemisodium salt (MES), H_2SO_4, trifluoroacetic Acid (TFA), acetone HPLC grade, 2-(4-(2-hydroxyethyl)piperazine-1-yl) ethane sulfonic acid (HEPES), poly(vinyl alcohol) (PVA), paraformaldehyde (PFA), and 6-diamidino-2-phenylindole dihydrochloride (DAPI) were purchased from Merck KGaA (DE). Galunisertib (LY 2157299) was purchased from Axon Medchem (NL). Recombinant protein-A and the mouse monoclonal L1-CAM antibody were purchased from Biotechne (IT). Hydroxypropyl methyl cellulose acetate succinate (HPMC-AS, herein abbreviated as HPMC) was purchased from Shin-Etsu Chemicam Co., Japan. Fasted state simulated intestinal fluid (FaSSIF) was purchased from Biorelevant.com Ltd (UK). Phosphate-buffered saline (PBS), non-essential aminoacids (NEEA), L-glutamine 200 mM, penicillin-streptomycin (Pen-Strep, 100 U mL^{-1}), sodium pyruvate (100 mM) and trypsin 2.5% were purchased from HyCloneTM, GE Healthcare Lifesciences (USA). CellMask ™ Deep Red, Hank's Balanced Salt Solution (HBSS), fetal bovine serum (FBS), ethylenediaminetetraacetic acid (EDTA) and Dulbecco's Modified Eagle Medium High Glucose (DMEM) were purchased from Life Technologies Gibco® (Waltham, MA, USA). Alexa Fluor® 488 was purchased from ThermoFisher Scientific (USA). Cell-Titer-Glo® reagent assay was purchased from Promega Corporation (Fitchburg, WI, USA). The 24, 96-well microplates, 25 cm^2 and 75 cm^2 cell culture flasks were purchased from Corning® Inc. (USA). Culture-inserts 2 well in μ-dishes 35 mm for migration assays were purchased from Ibidi (Giardini, IT).

2. Sonication, purification, and production of aminosilanized DNPs

For the production of DNPs, the diatomite powder was dispersed in ethanol and ultrasonicated with a Tip-Sonicator (Sonics Materials VC 750) by 40% amplitude (750 Watt and 20 kHz). The obtained DNPs were purified by a piranha solution made of a 4:1 mixture of concentrated H_2SO_4 and 30% wt. H_2O_2, and HCl to remove organic and metal contaminants, respectively. The purified DNPs were extensively washed with H_2O and collected by centrifugation (15000 rpm). Afterwards, DNPs were suspended in a 10% v/v APTES-ethanol solution for 1 hour at room temperature (RT) under mild stirring (400 rpm), collected, washed and characterized.

3. Fabrication of the microfluidic platform for the encapsulation of DNPs-Gel-Ab in HPMC

For the encapsulation of the DNPs-Gel-Ab, a borosilicate glass capillary with a diameter of 1.0 mm (World Precision Instruments, Inc., USA) was tapered using a micropipette puller (P-97, Sutter Instrument Co., USA) to a diameter of 20 µm. The inner diameter (d_i) of the capillary was further enlarged to ca. 100 µm. The inner capillary was inserted into an outer capillary (d_i = 1.1 mm, Sutter Instrument Co., USA) and coaxially aligned. Two polyethylene tubes were attached to the microfluidic chip to flush the inner and outer phases at constant flow rates separately. A three-dimensional (3D) coaxial flow was achieved by simultaneously pumping the inner and outer solutions in the same direction at different flow rates controlled by two pumps (PHD 2000, Harvard Apparatus). We tested different flow rate ratios of the inner phase (a dispersion of modified-DNPs in HPMC) and outer phase (PVA) to investigate their effects on the size, surface charge, and PDI of the formulation.

Flow rates ratio (mL h^{-1})	Size (nm)	Z-potential (mV)	PDI
1:15	450 ± 20	–15 ± 4	0.32 ± 0.02
1:20	540 ± 20	–16 ± 1	0.35 ± 0.05
1:30	320 ± 10	–20 ± 1	0.25 ± 0.01
1:40	270 ± 8	–25 ± 1	0.1 ± 0.03
1:60	220	–30 ± 2	0.2 ± 0.05

Table S1. Encapsulation of DNPs-Gel-Ab in HPMC using different flow rate ratios of inner and outer phases, respectively. The formulations obtained with lower flow rate ratios (1:15, 1:20) were characterized by increased size and PDI values, suggesting the formation of aggregates within the dispersion and a low encapsulation efficiency. Higher flow rate ratios (1:40 and 1:60), instead, caused the production of HPMC NPs rather than the precipitation of HPMC around modified-DNPs, as suggested by the tiny size and highly negative surface charge of the HPMC NPs. Using the flow rate ratio 1:30 (green row) we achieved a high encapsulation efficiency and obtained encapsulated-DNPs with good size, PDI, and morphology.

4. Stability studies of encapsulated-DNPs

The stability of the formulation in water-based solutions was evaluated by immersing the encapsulated-DNPs 1 mg mL^{-1} in MES buffer 10 mM pH 4.5 for 36 h at RT. The HPMC coating on the encapsulated-DNPs started dissolving at pH between 5.5 and 6.5, therefore MES pH 4.5 was selected as a buffer to avoid HPMC dissolution. For the stability studies, 0.05 mg of encapsulated-DNPs were taken from the dispersion at different times (0, 6, 12, 24, and 36 h) and analyzed by Dynamic Light Scattering (DLS) at the final concentration of 0.05 mg mL^{-1} (**Figure S1**). The developed formulation was stable in the buffer up to 36 h as both the size and PDI (**Figure S1 A**) did not change in the investigated time range. The PDI of encapsulated-DNPs was still 0.25 and the surface charge (**Figure S1 B**) was not altered after 36 h of incubation.

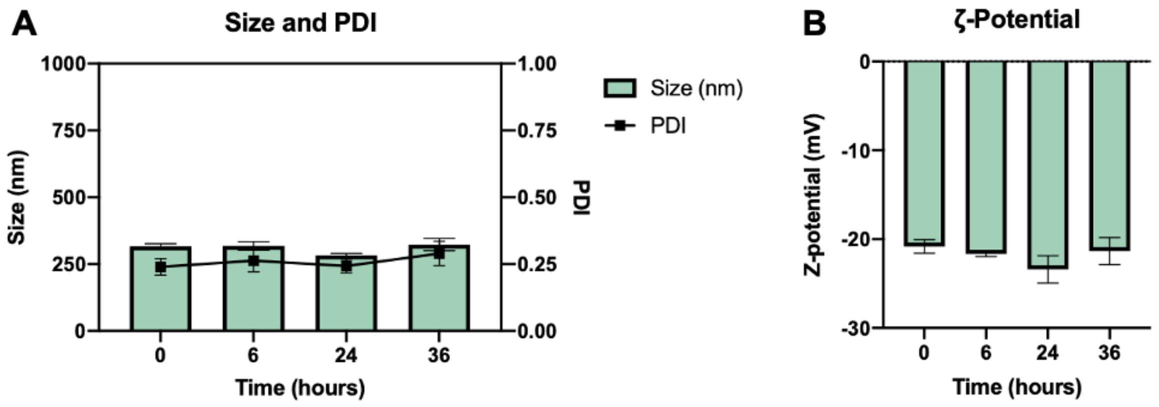

Figure S1. Stability of encapsulated-DNPs. (A) Size and PDI and (B) ζ-potential of encapsulated-DNPs dispersed in MES buffer pH 4.5 for 36 h at RT.

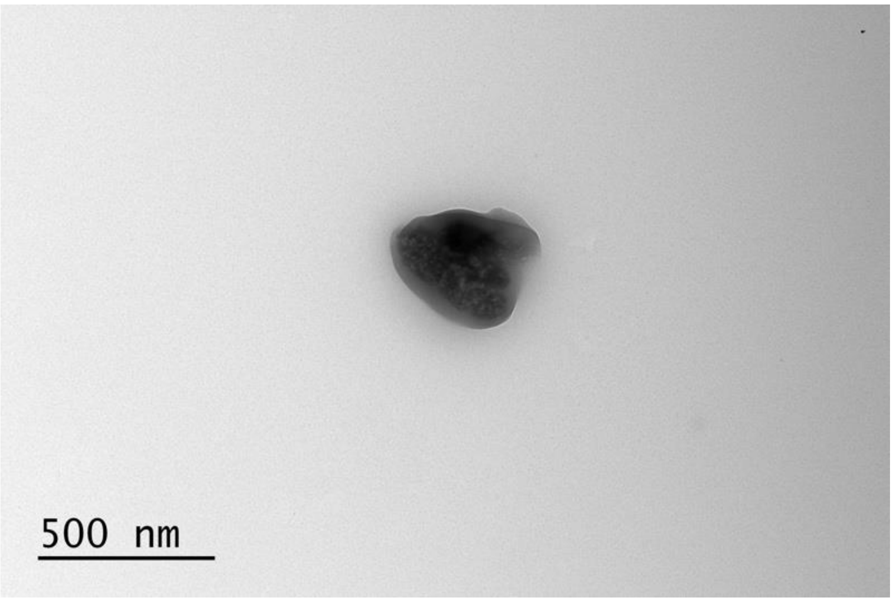

Figure S2. TEM image of the encapsulated-DNP obtained using the flow rate ratio 1:30. The microfluidic technology promoted the nanoprecipitation of the polymer matrix and the formation of a homogeneous layer around the encapsulated-DNP. Here, the outer polymer layer and the siliceous DNP inside can be distinguished.

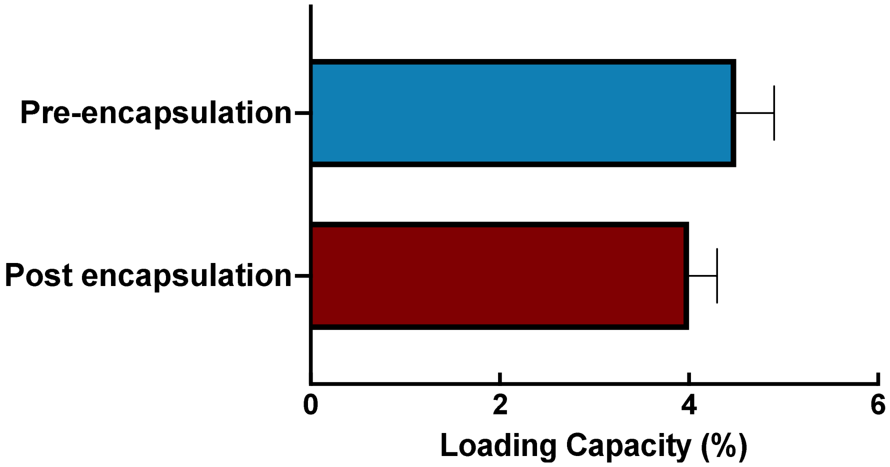

Figure S3. Drug loading capacity of the DNPs-Gel-Ab (pre-encapsulation) and encapsulated-DNPs investigated by RP-HPLC. Results are expressed as mean ± standard deviation (n=3).

The loading capacity of the encapsulated-DNPs decreased by 11% after the microfluidic encapsulation, due to the loss of drug absorbed on the surface of the DNP-Gel-Ab.

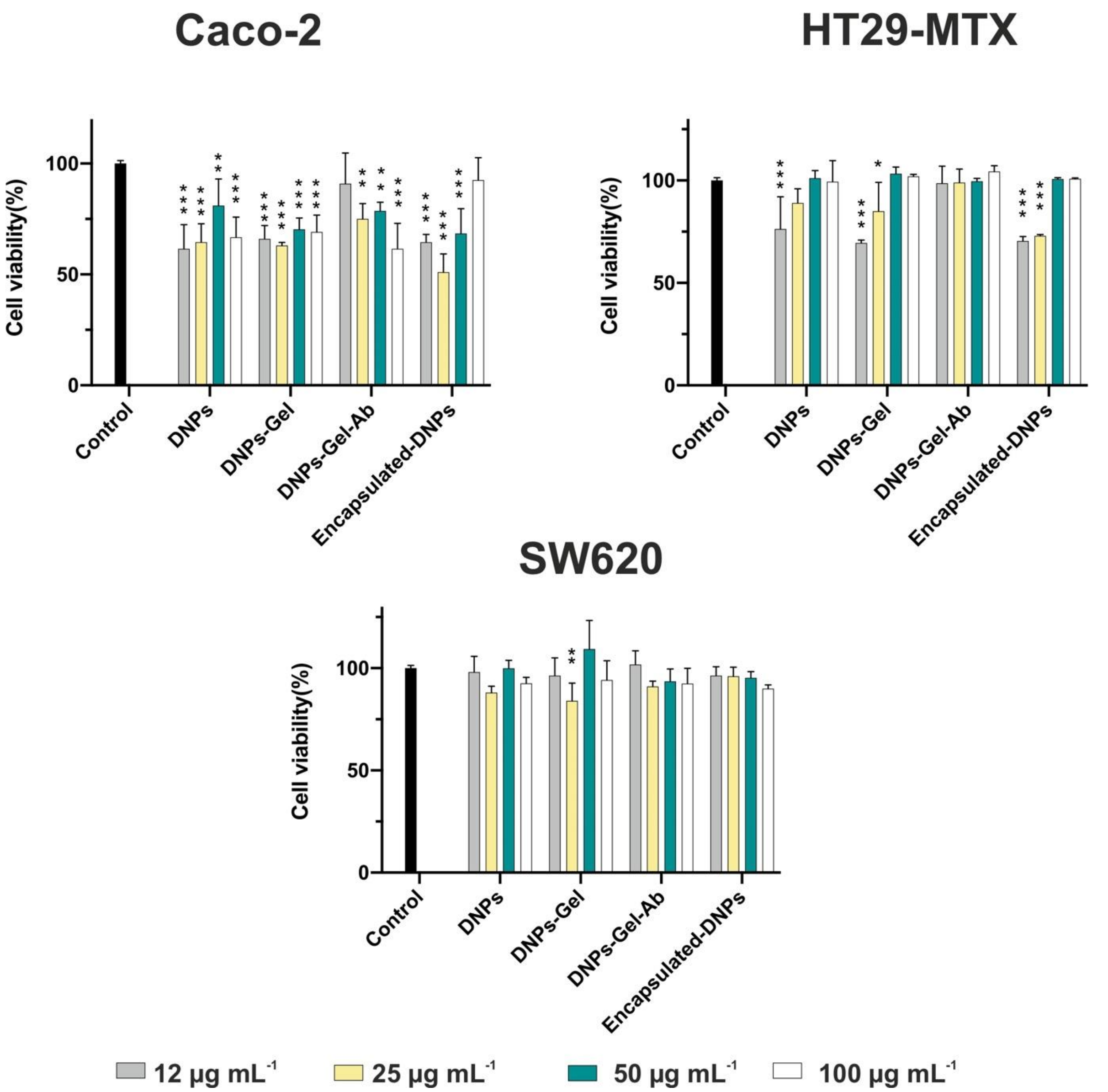

Figure S4. Biocompatibility studies. Cell viability (%) of Caco-2, HT29-MTX, and SW620 incubated with NPs at different steps of preparation (DNPs, DNPs-Gel, DNPs-Gel-Ab, encapsulated-DNPs) and concentrations ranging from 25 to 100 μg mL^{-1} for 72 h. Cells were incubated with HBSS−HEPES buffer (pH 7.4) as the negative control. Each data set was compared to the negative control. The level of significance was set at probabilities of * $p < 0.05$, ** $p < 0.01$ and *** $p < 0.001$. Non-significant results are not reported. Results are expressed as mean ± s.d. ($n \geq 3$).

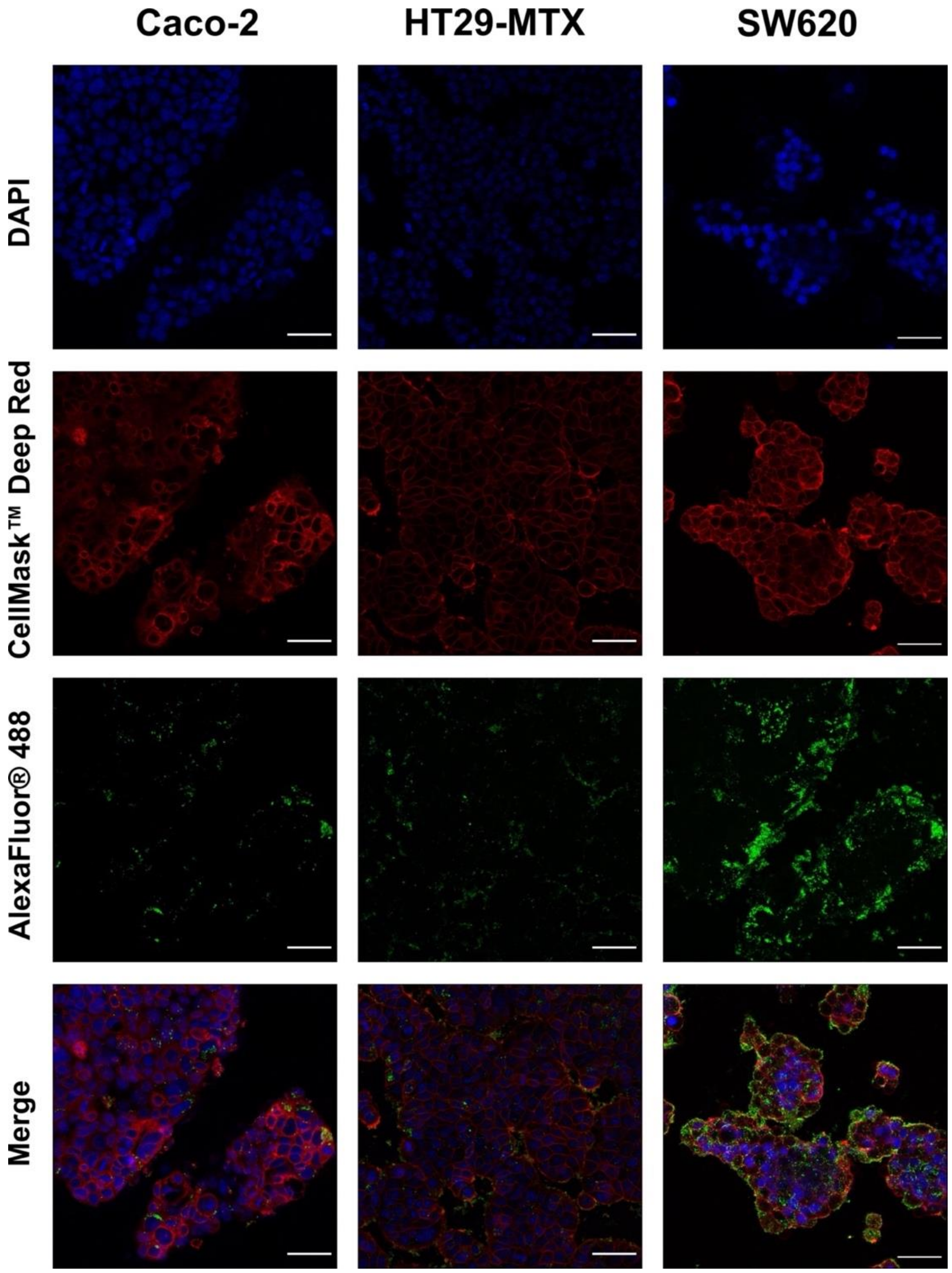

Figure S5. Interactions of encapsulated-DNPs with different cell lines. Confocal fluorescence microscopy of Caco-2, HT29-MTX, and SW620 cells after incubation with 50 μg of encapsulated-DNPs for 24 h at 37 °C in PBS. CellMask ™ Deep Red (red) was used to stain the cell membranes; the encapsulated-DNPs were labeled with Alexa Fluor ®-488 (green); nuclei were stained with DAPI (blue). The scale bar is 50 μm. The images were acquired with a Leica SP8 microscope using a 63× objective.
